# Supplementary material for: Condensation of the fusion focus by the intrinsically disordered region of the formin Fus1 is essential for cell-cell fusion
Source: Curr Biol. 2022 Nov 7;32(21):4752–4761.e10. doi: 10.1016/j.cub.2022.09.026 (PMC9671092; doi:10.1016/j.cub.2022.09.026)
Supplement: Document S1. Figures S1 and S2 and Tables S1–S3 [file mmc1.pdf]

Current Biology, Volume *32*

Supplemental Information

Condensation of the fusion focus by the intrinsically disordered region of the formin Fus1 is essential for cell-cell fusion

Ingrid Billault-Chaumartin, Olivia Muriel, Laetitia Michon, and Sophie G. Martin

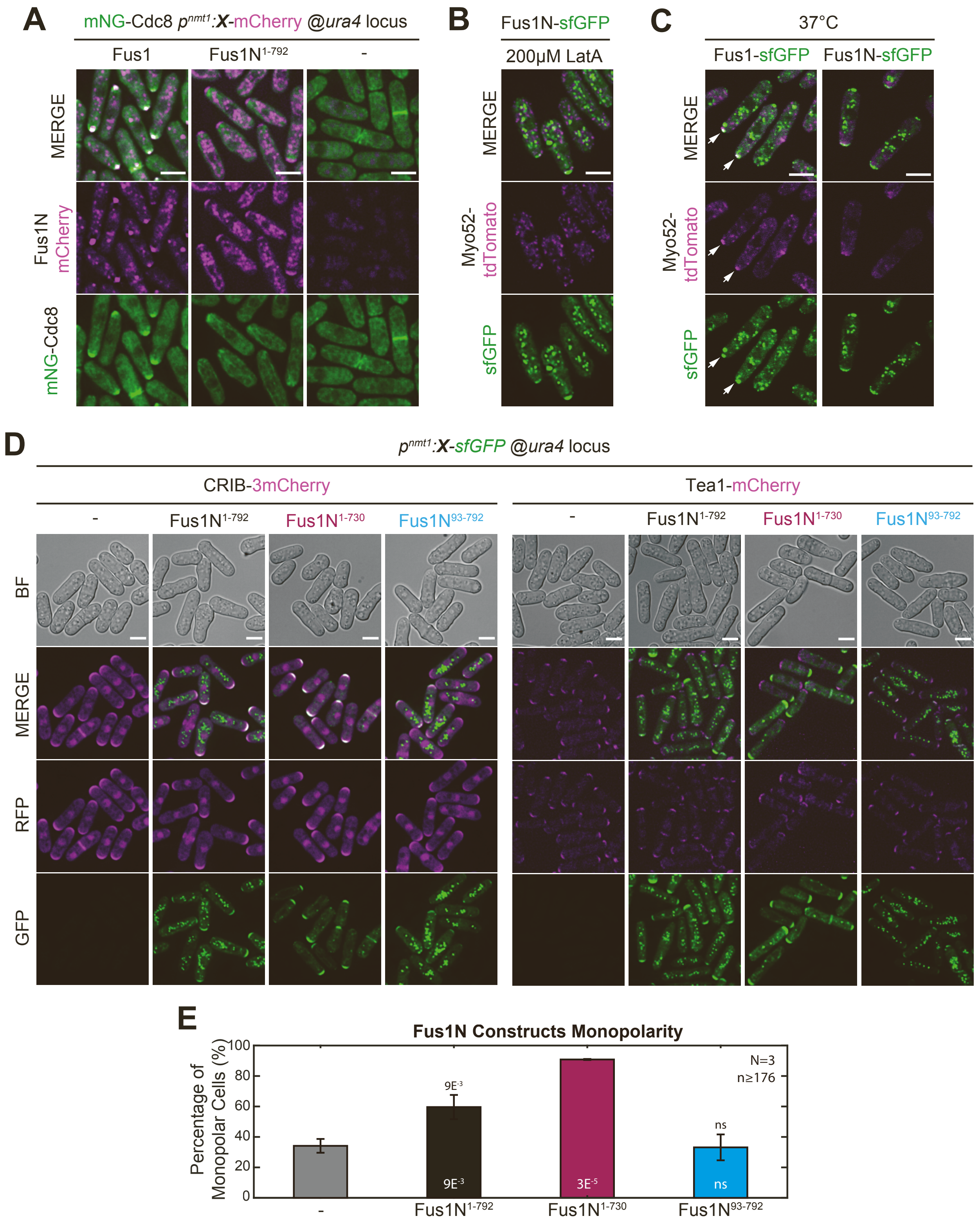

Figure S1 . Additional localization data and Fus1N tip localization effect on polarity. Related to Figure 1.

A. Interphase cells expressing mNeonGreen-Cdc8, either in combination with (left) full length Fus1-sfGFP or (middle) Fus1N-sfGFP (Fus1$^{1\text{-}792}$) or (right) alone. **B.** Interphase cells expressing Myo52-tdTomato and Fus1N-sfGFP (Fus1$^{1\text{-}792}$). Cells were treated with 200µM LatrunculinA for 5 minutes. **C.** Interphase cells expressing Myo52-tdTomato and either (left) full length Fus1-sfGFP or (right) Fus1N-sfGFP (Fus1$^{1\text{-}792}$) grown for 6h at 37°C and imaged at 40°C. White arrows mark resistant fusion focus-like structure. **D.** DIC

and fluorescence images of either (left) the polarity marker CRIB-3mCherry or (right) Tea1-mCherry in interphase WT or Fus1N$^{1-792}$, Fus1N$^{1-730}$ or Fus1N$^{93-792}$-expressing cells. **E.** Monopolarity of the strains as in (D), assessed from the localization of CRIB on a single snapshot. Of note, a fraction of WT cells appear monopolar using this assay, because they are either before NETO or at a time point in CRIB oscillations [52] where only one tip is decorated. All p-values are relative to WT. Bars are 5µm.

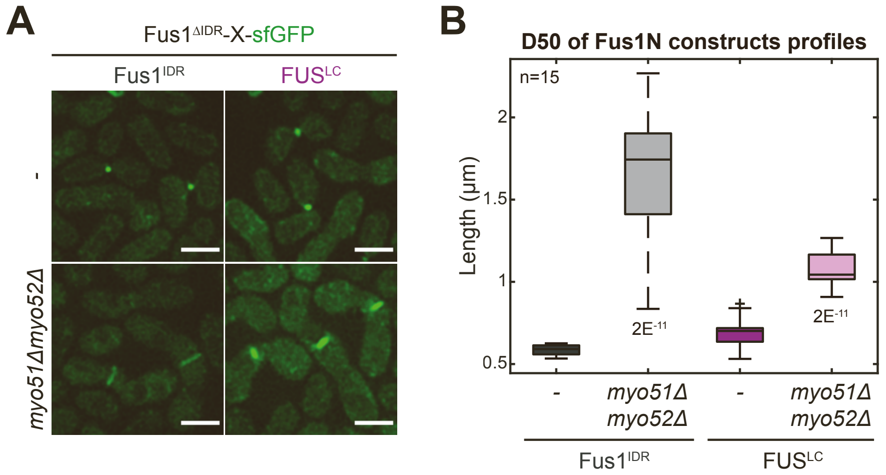

Figure S2. Type V myosins are required for fusion focus focalization in both WT cells and cells in which Fus1 IDR was replaced by FUS^{LC}. Related to Figure 4.

A. Images of (left) Fus1-sfGFP or (right) Fus1$^{1-491}$-FUS^{LC}-Fus1$^{792-1372}$-sfGFP in mating cell pairs that are otherwise WT (top) or carry deletions of *myo51Δ* and *myo52Δ* (bottom). **B.** Width at half maximum of GFP-fluorescence profiles in strains as in (A). p-values compare WT Fus1 and Fus1$^{1-491}$-FUS^{LC}-Fus1$^{792-1372}$ in each background. Bars are 5µm.

Table S1: Strains used in this study and links to figures. Related to STAR Methods.

GENOTYPE	FIGURES	STRAIN
h90 myo52-tdTomato:natMX fus1-sfGFP:kanMX ura4- leu1-32 ade6-M216	1, 4, S2	YSM3312
h90 myo52-tdTomato:natMX fus1Δ::LEU2+ ura4-294:p^{fus1}-fus1N^{1-792}-fus1$C^{793-1372}$-sfGFP:ura4+ leu1-32	1	YSM2504
h90 myo52-tdTomato:natMX fus1Δ::LEU2+ ura4-294:p^{fus1}-cdc12N^{1-887}-fus1$C^{793-1372}$-sfGFP:ura4+ leu1-32	1	YSM2512
h90 myo52-tdTomato:natMX fus1Δ::LEU2+ ura4-294:p^{fus1}-for3N^{1-714}-fus1$C^{793-1372}$-sfGFP:ura4+ leu1-32	1	YSM2510
h90 myo52-tdTomato:natMX ura4+:p^{nmt1}:fus1N^{1-792}-sfGFP:$term^{nmt}$ leu1-32 ade6-M210	1, 3, S1	YSM4002
h90 myo52-tdTomato:natMX ura4+:p^{nmt1}:fus1N^{1-730}-sfGFP:$term^{nmt}$ leu1-32 ade6-M210	1, 3	YSM4003
h90 myo52-tdTomato:natMX ura4+:p^{nmt1}:fus1N^{1-500}-sfGFP:$term^{nmt}$ leu1-32 ade6-M210	1, 3	YSM4004
h90 myo52-tdTomato:natMX ura4+:p^{nmt1}:fus1N^{93-792}-sfGFP:$term^{nmt}$ leu1-32 ade6-M210	1	YSM4005
h90 myo52-tdTomato:natMX ura4+:p^{nmt1}:fus1$N^{140-792}$-sfGFP:$term^{nmt}$ leu1-32 ade6-M210	1	YSM4006
h90 myo52-tdTomato:natMX ura4+:p^{nmt1}:fus1$N^{191-792}$-sfGFP:$term^{nmt}$ leu1-32 ade6-M210	1, 3	YSM4007
h90 myo52-tdTomato:natMX ura4+:p^{nmt1}:fus1$N^{431-755}$-sfGFP:$term^{nmt}$ leu1-32 ade6-M210	1	YSM4008
h90 myo52-tdTomato:natMX ura4+:p^{nmt1}:fus1-sfGFP:$term^{nmt}$ leu1-32 ade6-M210	1, S1	YSM4009
h90 leu1-32:p^{cdc8}:mNeonGreen-cdc8:$term^{cdc8}$:$term^{ScADH1}$:leu1+ ura4-D18 ade6-M216	S1	YSM3786
h90 ura4+:p^{nmt1}:fus1-mCherry:$term^{nmt}$ leu1-32:p^{cdc8}:mNeonGreen-cdc8:$term^{cdc8}$:$term^{ScADH1}$:leu1+ ade6-M216	S1	YSM4042
h90 ura4+:p^{nmt1}:fus1N^{1-792}-mCherry:$term^{nmt}$ leu1-32:pcdc8:mNeonGreen-cdc8:$term^{cdc8}$:$term^{ScADH1}$:leu1+ ade6-M216	S1	YSM4043
h+ his5+:p^{act1}:CRIB-3mCherry:bsdMX ura4-D18	S1	YSM4010
h90 his5+:p^{act1}:CRIB-3mCherry:bsdMX ura4+:p^{nmt1}:fus1N^{1-792}-sfGFP:$term^{nmt}$ leu1-32	S1	YSM4011
h90 his5+:p^{act1}:CRIB-3mCherry:bsdMX ura4+:p^{nmt1}:fus1N^{1-730}-sfGFP:$term^{nmt}$ ade6-M210	S1	YSM4012
h90 his5+:p^{act1}:CRIB-3mCherry:bsdMX ura4+:p^{nmt1}:fus1N^{93-792}-sfGFP:$term^{nmt}$ leu1-32 ade6-M210	S1	YSM4013
h90 tea1-mCherry:kanMX ura4-D18 leu1-32	S1	YSM4014
h90 tea1-mCherry:kanMX ura4+:p^{nmt1}:fus1N^{1-792}-sfGFP:$term^{nmt}$ leu1-32	S1	YSM4015
h90 tea1-mCherry:kanMX ura4+:p^{nmt1}:fus1N^{1-730}-sfGFP:$term^{nmt}$ leu1-32	S1	YSM4016
h90 tea1-mCherry:kanMX ura4+:p^{nmt1}:fus1N^{93-792}-sfGFP:$term^{nmt}$ leu1-32 ade6-M210	S1	YSM4017
h90 myo52-tdTomato:natMX fus1-sfGFP:kanMX	2	YSM3888
h90 myo52-tdTomato:natMX fus1-sfGFP:kanMX acp2Δ::bleMX ura4- leu1-32 ade6-M210	2	YSM3314
h90 myo52-tdTomato:natMX ura4+:p^{nmt1}:fus1N^{93-792}-sfGFP:$term^{nmt}$ fus1Δ::hphMX ade6-M210 leu1-32	2	YSM4018
h90 myo52-tdTomato:natMX ura4+:p^{nmt1}:fus1-sfGFP:$term^{nmt}$ fus1Δ::hphMX ade6-M210 leu1-32	2	YSM4053
h90 myo52-tdTomato:natMX ura4-294:p^{fus1}:fus1N-sfGFP:ura4+ fus1Δ::LEU2+ leu1-32	3	YSM2486
h90 myo52-tdTomato:natMX ura4-294:p^{fus1}:fus1N-sfGFP:ura4+ leu1-32	3	YSM2699
h90 myo52-tdTomato:natMX ura4+:p^{nmt1}:fus1N^{1-792}-sfGFP:$term^{nmt}$ fus1Δ::hphMX leu1-32 ade6-M210	3	YSM4054
h90 myo52-tdTomato:natMX ura4+:p^{nmt1}:fus1$N^{191-792}$-sfGFP:$term^{nmt}$ fus1Δ::hphMX leu1-32 ade6-M210	3	YSM4055
h90 myo52-tdTomato:natMX ura4+:p^{nmt1}:fus1N^{1-730}-sfGFP:$term^{nmt}$ fus1Δ::hphMX leu1-32 ade6-M210	3	YSM4056
h90 myo52-tdTomato:natMX ura4+:p^{nmt1}:fus1N^{1-500}-sfGFP:$term^{nmt}$ fus1Δ::hphMX leu1-32 ade6-M210	3	YSM4057
h90 myo52-tdTomato:natMX fus1$^{\Delta 501-749}$-sfGFP:kanMX ura4-294 leu1-32 ade6-M210	3	YSM4019
h90 myo52-tdTomato:natMX fus1$^{\Delta 501-791}$-sfGFP:kanMX ura4-294 leu1-32 ade6-M210	3	YSM4020
h90 myo52-tdTomato:natMX fus1$^{\Delta 492-791}$-sfGFP:kanMX ura4-294 leu1-32 ade6-M210	3, 4	YSM4021
h90 myo52-tdTomato:natMX fus1$^{\Delta 492-500}$-sfGFP:kanMX ura4-294 leu1-32 ade6-M210	3	YSM4044
h90 myo52-tdTomato:natMX fus1$^{\Delta 731-791}$-sfGFP:kanMX ura4-294 leu1-32 ade6-M210	3	YSM4045
h90 myo52-tdTomato:natMX fus1$^{\Delta 492-749}$-sfGFP:kanMX ura4-294 leu1-32 ade6-M210	3	YSM4046
h90 myo52-tdTomato:natMX fus1$^{1-491}$-FUS^{12E}-fus1$^{792-1372}$-sfGFP:kanMX ura4-294 leu1-32 ade6-M210	4	YSM4022
h90 myo52-tdTomato:natMX fus1$^{1-491}$-FUS-fus1$^{792-1372}$-sfGFP:kanMX ura4-294 leu1-32 ade6-M210	4, S2	YSM4023
h90 myo52-tdTomato:natMX fus1$^{1-491}$-$CRY2^{PHR}$-fus1$^{792-1372}$-sfGFP:kanMX ura4-294 leu1-32 ade6-M210	4	YSM4024
h90 myo52-tdTomato:natMX fus1$^{1-491}$-$CRY2^{olig}$-fus1$^{792-1372}$-sfGFP:kanMX ura4-294 leu1-32 ade6-M210	4	YSM4025
h90 myo52-tdTomato:natMX fus1$^{1-491}$-FUS^{G156E}-fus1$^{792-1372}$-sfGFP:kanMX ura4-294 leu1-32 ade6-M210	4	YSM4047
h90 myo52-tdTomato:natMX leu1-32:p^{cdc8}:mNeonGreen-cdc8:$term^{cdc8}$:$term^{ScADH1}$:leu1+ fus1:kanMX ura4-294 ade6-M210	4	YSM4026
h90 myo52-tdTomato:natMX leu1-32:p^{cdc8}:mNeonGreen-cdc8:$term^{cdc8}$:$term^{ScADH1}$:leu1+ fus1$^{1-491}$-$CRY2^{PHR}$-fus1$^{792-1372}$:kanMX ura4-294 ade6-M210	4	YSM4048
h90 myo52-tdTomato:natMX leu1-32:p^{cdc8}:mNeonGreen-cdc8:$term^{cdc8}$:$term^{ScADH1}$:leu1+ fus1$^{1-491}$-$CRY2^{olig}$-fus1$^{792-1372}$:kanMX ura4-294 ade6-M210	4	YSM4049
h90 myo52-tdTomato:natMX leu1-32:p^{cdc8}:mNeonGreen-cdc8:$term^{cdc8}$:$term^{ScADH1}$:leu1+ fus1$^{1-491}$-FUS-fus1$^{792-1372}$:kanMX ura4-294 ade6-M210	4	YSM4050
h90 myo52-tdTomato:natMX leu1-32:p^{cdc8}:mNeonGreen-cdc8:$term^{cdc8}$:$term^{ScADH1}$:leu1+ fus1$^{1-491}$-FUS^{12E}-fus1$^{792-1372}$:kanMX ura4-294 ade6-M210	4	YSM4051
h90 fus1-sfGFP:kanMX myo51Δ::ura4+ myo52Δ::ura4+ leu1-32	S2	YSM2543
h90 fus1$^{1-491}$-FUS-fus1$^{792-1372}$-sfGFP:kanMX myo51Δ::ura4+ myo52Δ::ura4+ leu1-32	S2	YSM4052

Table S2: Primers used in this study and their usage

HR stands for homologous recombination in yeast [45] and SDM for site directed mutagenesis

NAME	SEQUENCE	ORIENTATION	PURPOSE
osm765	CAGCTCCAAATTTTGAAAGTAAAACCCCTAATTAGGGAATAAATAAGTAGGCAGAGCAC CTTGAAAAAATAACTAGATAGAATTCGAGCTCGTTTAAAC	R	HR (Myo52 3')
osm932	AATAAAAAGAGACAAACAGTCGTCCTTAAAGCTGAATGCATGCTTAAGCAGCTGGAGA ATAACAATGAACTTAAGAGACGGATCCCCGGGTTAATTAA	F	HR (Fus1 ORF)
osm933	TTTTATTAATTATAATTTCATTATAATTTGTTTAAGTCATTTAATTGTCATTAAAAGTCATT AACATTTCAAACATCAGAATTCGAGCTCGTTTAAAC	R	HR (Fus1 3')
osm1196	GATCACTGTAGGCAACGTAGCCGACAATGATGTACAGAACTCGAGCGACGAAGAAAAT CAAGTACCAAATGGTATTAAAGTTCGGATCCCCGGGTTAATTAA	F	HR (Myo52 ORF)
osm1746	ACGGATTTCATGAAGTTATTGGTTAAAAGCGGCCTCTCAAATCCTCCAGCTAAAGAACCA GTCCATGACAACGAAAATCGGATCCCCGGGTTAATTAA	F	HR (Tea1 ORF)
osm1747	ATGTCATCGTCGAATATTTACACTATGTACAGTCCTTTCAACTAGTAAAGGAGATGCTTT CAAAATAGTTCCAAAGAGGAATTCGAGCTCGTTTAAAC	R	HR (Tea1 3')
osm1772	CGTATCACGAGGCCCTTTCG	F	CLONING
osm2217	CCGGATCCTCCAAGGGTGAAGAGCTATTTACTGGGG	F	CLONING
osm3005	ACTGCGGCCGCATGATGACGGCTAGTTTTAAAGG	F	CLONING
osm3006	ACTCCCGGGTCTCTTAAGTTCATTGTTATTCTCC	R	CLONING
osm3007	ACTGCGGCCGCATGGCATCTAAAATGCCTGAAG	F	CLONING
osm3009	ACTGCGGCCGCATGCGAAATTCGTCAAAGGGAC	F	CLONING
osm3026	CTTGGATCCTCATATTTTCTATTTTAGAAAACCTC	R	CLONING
osm3027	TGAGGATCCAAGAAGTTATTGATGGGAATCC	F	CLONING
osm3028	CTGGGATCCATGGCGAAGGCGAGGAAG	R	CLONING
osm3030	TCGGGATCCTACTATTGTTGCTAACTGTTTCTGC	R	CLONING
osm3031	GTAGGATCCCGAACTTTGATATTCCTAATGATGC	F	CLONING
osm3091	CGGGGTACCGATCAGAAAATTATCGCCAT	F	CLONING
osm3516	ACTGCGGCCGCTGATTTAACAAAGCGACTATAAGTC	R	CLONING
osm3521	ACTCCCGGGAGTAGAAGTGTTAGGAGCTTC	R	CLONING
osm4021	CTTGGATCCTATGAACCTCAAAAGAATGCGTTG	R	CLONING
osm4504	CATTAAGGCCTCACTTTTATTCTGAGATCGCTATCCGGTTGTATTCTTTTGTTTAAAGCAT TATATCATCAACTCACCCGGATCCCCGGGTTAATTAA	F	HR (Acp2 5')
osm4505	CAATCTTTCTATGACTATTTTCGTTGAAGATGGAACGAATACTATGAGAAGATCACGGAA AGAAAACAAAAAGCAATCGAATTCGAGCTCGTTTAAAC	R	HR (Acp2 3')
osm4577	GGAATAAGGGCGACACGG	F	ANALYTICAL
osm5452	GGCCACTAGTGGATCTGATATCGATGTATTTACTGATTACTT	R	INFUSION
osm5453	CTTCTAAACGGCTAGCTCAGCTTCATTGG	F	SDM
osm5454	CAATGAAGCTGAGCTAGCCGTTTAGAAGG	R	SDM
osm6064	CATATGGTCTGGGTATCT	R	CLONING
osm6183	GCCTTCCAACCAGCTTCTCT	R	ANALYTICAL
osm6576	CTTGGATCCATCATTATTTGAATTACCAT	R	CLONING
osm6582	CTTGTTTAAACCAACATGCCTGTAAG	R	CLONING
osm6583	GAAGTTTAAACTGCTTTTGTGGTTATC	F	CLONING
osm7119	CTTCGTACGCTGCAGGTCGACACAGTATGTACGCCAC	F	INFUSION
osm7122	TTCACCCTTGGAGTTAATTAATCTCTTAAGTTCATTGTTAT	R	INFUSION
osm7127	ATGTACCAGGCGAAGCGCTTCTATGTCCGGATGAC	F	INFUSION
osm7140	CTTCTTTGATTCTCATATCAGCTTGTAAAGTAAGC	R	INFUSION
osm7141	TACTTTACAAGCTGATATGAGAATCAAAGAAGTTAT	F	INFUSION
osm7204	CTTTGTTAAATCAGCGGCCGCATGTTTACCGATTCATATGTA	F	INFUSION
osm7205	CTTGGAGTTAATTAACCCGGGGATCCTCATATTTTC	R	INFUSION
osm7254	GCTTTGTTAAATCAGCGGCCGCATGATGAC	F	INFUSION
osm7255	CTTGGAGTTAATTAACCCGGGGATCCTATCATTATTTGAATTACCA	R	INFUSION
osm7256	CTTTGTTAAATCAGCGGCCGCATGAAGCACACTCCAAATTCT	F	INFUSION
osm7257	CTTGGAGTTAATTAACCCGGGGATCCTAAAAACCTTGTGTTTTGA	R	INFUSION
osm7487	CTTCTTTGATTCTCATATCATTATTTGAATTACCAT	R	INFUSION
osm7488	TAATTCAAATAATGATATGAGAATCAAAGAAGTTAT	F	INFUSION
osm7489	AAACCTTGTGTTTTGAATCAGCTTGTAAAGTAAG	R	INFUSION
osm7490	TACTTTACAAGCTGATTCAAAACACAAGGTTTTTA	F	INFUSION
osm7499	GCTTTGTTAAATCAGCGGCCGCATGCTCAAGTACGTGGAATCTTT	F	INFUSION

osm7638	CTTTGTTAAATCAGCGGCCGCATGGTTACACTCTCTCAAGAAAA	F	INFUSION
osm7677	GGAGTATTAAAACAACTCGAGAAATGCGTGAAACTC	F	INFUSION
osm7690	AAATCAAGGATATGAGAATTCCGAAAGAAAGTATGT	F	INFUSION
osm7738	TATAAAAGCAATCAATATCAGCTTGTAAAGTAAGCAC	R	INFUSION
osm7739	TACTTTACAAGCTGATATTGATTGCTTTTATAAGGAATTAAAG	F	INFUSION
osm7740	GCTTATTTAGAAGTGGCGCGCCTCTCTTAAGTTCATTGTTATTC	R	INFUSION
osm7875	CTTCTTTGATTCTCATATGAACCTCAAAAGAATGCG	R	INFUSION
osm7876	TTCTTTTGAGGTTCATATGAGAATCAAAGAAGTTATTGAT	F	INFUSION
osm7877	CTTCTGATTTACAGTGCTAGCCTTTTTGTACTCCAGTATTAT	R	INFUSION
osm7878	TTTTGTCCATCTTCATCGTCATCATTAACAAGCAATAG	R	INFUSION
osm7879	CTTGTTAATGATGACGATGAAGATGGACAAAAAGACTAT	F	INFUSION
osm7880	AACTAGCCGTCATCATTGCTGCTCCGATCATGATCT	R	INFUSION
osm7881	CATGATCGGAGCAGCAATGATGACGGCTAGTTTTAAAG	F	INFUSION
osm7882	GAGTTTCACGCATTTCTCGAGTTGTTTTAATACTCCTTC	R	INFUSION
osm8388	ACTGGTTCTGCTGTTCATAGCCCTGAGGGGGATTA	R	INFUSION
osm8389	CCCTCAGGGCTATGAACAGCAGAACCAGTACAAC	F	INFUSION
osm8480	ACTTAAGAGAGGATCCCCGGGTTAATTAAC	F	INFUSION
osm8481	ATTCCTTTTACCCGGTTTACTTGTACAGCTCGTCC	R	INFUSION
osm8482	CGAGCTGTACAAGTAAACCGGGTAAAAGGAATGTC	F	INFUSION
osm8483	AGGGAACAAAAGCTGGAGC	R	INFUSION
osm8484	GAAAATATGAGGATCCCCGGGTTAATTAAC	F	INFUSION

Table S3: Plasmids used in this study and their construction. Related to STAR Methods.

For each plasmid, the column “obtained from” indicates how it was constructed, from restriction enzyme-based cloning or infusion, with the primers and restriction enzymes used. “WT” indicates that genomic DNA from a wildtype strain was used as template for PCR amplification.

NAME	DESCRIPTION	OBTAINED FROM	USAGE
pAV133	pUra4^{AfeI}	[46]	Single integration at *ura4*
pSM617	pREP3x	Lab Stock	Pombe expression
pSM677	pFA6a-mCherry-kanMX	Lab Stock	template for PCR-based HR
pSM684	pFA6a-mCherry-natMX	Lab Stock	template for PCR-based HR
pSM685	pFA6a-tdTomato-natMX	Lab Stock	template for PCR-based HR
pSM694	pFA6a-bleMX	Lab Stock	template for PCR-based HR
pSM1538	pFA6a-sfGFP-kanMX	Lab Stock	template for PCR-based HR
pSM1638	pRIP-p^{fus1}-sfGFP	Lab Stock	Multiple integration at *ura4*
pSM1650	pRIP-p^{fus1}-fus1N-sfGFP	Regular cloning : pSM1638$^{NotI/BamHI}$+(WT$^{osm3005-osm3026}$)$^{NotI/BamHI}$	Multiple integration at *ura4*
pSM1656	pRIP-p^{fus1}-fus1-sfGFP	Regular cloning : pSM1638$^{NotI/XmaI}$+(WT$^{osm3005-osm3006}$)$^{NotI/XmaI}$	Multiple integration at *ura4*
pSM1659	pRIP-p^{fus1}-fus1N-fus1C-sfGFP	3-point ligation cloning : pSM1638$^{NotI/XmaI}$+(WT$^{osm3005-osm3026}$)$^{NotI/BamHI}$ +(WT$^{osm3027-osm3006}$)$^{BamHI/XmaI}$	Multiple integration at *ura4*
pSM1662	pRIP-p^{fus1}-for3N-fus1C-sfGFP	3-point ligation cloning : pSM1638$^{NotI/XmaI}$+(WT$^{osm3007-osm3028}$)$^{NotI/BamHI}$ +(WT$^{osm3027-osm3006}$)$^{BamHI/XmaI}$	Multiple integration at *ura4*
pSM1663	pRIP-p^{fus1}-cdc12N-fus1C-sfGFP	3-point ligation cloning : pSM1638$^{NotI/XmaI}$+(WT$^{osm3009-osm3030}$)$^{NotI/BamHI}$ +(WT$^{osm3027-osm3006}$)$^{BamHI/XmaI}$	Multiple integration at *ura4*
pSM1823	pRIP-p^{nmt41}-sfGFP	Lab Stock	Multiple integration at *ura4*
pSM1826	pRIP-p^{nmt41}-fus1N-sfGFP	Subcloning : pSM1650$^{KpnI/NotI}$+pSM1823$^{KpnI/NotI}$	Multiple integration at *ura4*
pSM2229	pUra4^{AfeI}-p^{nmt41}-fus1-sfGFP	Lab Stock, Derived from pAV133	Single integration at *ura4*
pSM2251	pFA6a-fus1$^{5'UTR}$-fus1_K879A-sfGFP-kanMX-fus1$^{3'UTR}$	SDM : pSM2827$^{osm5453/5454}$	Single integration at endogenous *fus1* locus
pSM2390	pRIP-p^{fus1}-CRY2olig-For3N-fus1C-sfGFP	Lab Stock, Derived from pAV133	Multiple integration at *ura4*
pSM2475	pUra4^{AfeI}-p^{fus1}-CRY2PHR-fus1C-sfGFP	Lab Stock, Derived from pSM1662	Single integration at *ura4*
pSM2478	pUra4^{PmeI}-p^{nmt41}-fus1-sfGFP	3-point ligation cloning : pSM2229$^{AatII/StuI}$+(pSM2229$^{osm4577-osm6582}$)$^{AatII/PmeI}$ +(pSM2229$^{osm6583-osm6183}$)$^{PmeI/StuI}$	Single integration at *ura4*
pSM2507	pFA6a-fus1$^{5'UTR}$-fus1$^{\Delta 501-749}$-sfGFP-kanMX-fus1$^{3'UTR}$	3-point ligation cloning : pSM2251$^{SalI/PacI}$+(pSM2251$^{osm1772-osm6576}$)$^{SalI/BamHI}$ +(pSM2229$^{osm3031-osm3521}$)$^{BamHI/PacI}$	Single integration at endogenous *fus1* locus
pSM2600	pUra4^{PmeI}-p^{nmt1}-fus1N-sfGFP	3-point ligation cloning : pSM2478$^{KpnI/SacI}$+(pSM617$^{osm3091-osm3516}$)$^{KpnI/NotI}$ +pSM1826$^{NotI/SacI}$	Single integration at ura4
pSM2601	pUra4^{PmeI}-p^{nmt1}-fus1N$^{1-730}$-sfGFP	3-point ligation cloning: pSM2600$^{NotI/MscI}$+(pSM2600$^{osm3005-osm4021}$)$^{NotI/BamHI}$+(pSM2600$^{osm2217-osm6064}$)$^{BamHI/MscI}$	Single integration at *ura4*
pSM2602	pUra4^{PmeI}-p^{nmt1}-fus1-sfGFP	Subcloning : pSM2600$^{NotI/XmaI}$+pSM1656$^{NotI/XmaI}$	Single integration at *ura4*
pSM2625	pFA6a-fus1$^{5'UTR}$-fus1$^{\Delta 492-791}$-sfGFP-kanMX-fus1$^{3'UTR}$	Infusion cloning : pSM2507$^{SalI/PacI}$+WT$^{osm7119-osm7140}$ +WT$^{osm7141-osm7122}$	Single integration at endogenous *fus1* locus
pSM2630	pUra4^{PmeI}-p^{nmt1}-fus1N$^{93-792}$-sfGFP	Infusion cloning : pSM2600$^{NotI/XmaI}$+pSM2600$^{osm7204-osm7205}$	Single integration at *ura4*
pSM2644	pUra4^{PmeI}-p^{nmt1}-fus1N$^{1-500}$-sfGFP	Infusion cloning : pSM2600$^{NotI/XmaI}$+pSM2600$^{osm7254-osm7255}$	Single integration at *ura4*
pSM2645	pUra4^{PmeI}-p^{nmt1}-fus1N$^{431-755}$-sfGFP	Infusion cloning : pSM2600$^{NotI/XmaI}$+pSM2600$^{osm7256-osm7257}$	Single integration at *ura4*

pSM2697	pFA6a-fus1$^{5'UTR}$-fus1$^{\Delta 501\text{-}791}$-sfGFP-kanMX-fus1$^{3'UTR}$	Infusion cloning : pSM2507$^{SalI/PacI}$+WT$^{osm7119\text{-}osm7487}$ +WT$^{osm7488\text{-}osm7122}$	Single integration at endogenous *fus1* locus
pSM2698	pFA6a-fus1$^{5'UTR}$-fus1$^{\Delta 492\text{-}749}$-sfGFP-kanMX-fus1$^{3'UTR}$	Infusion cloning : pSM2507$^{SalI/PacI}$+WT$^{osm7119\text{-}osm7489}$ +WT$^{osm7490\text{-}osm7122}$	Single integration at endogenous *fus1* locus
pSM2703	pUra4^{PmeI}-p^{nmt1}-fus1N$^{191\text{-}792}$-sfGFP	Infusion cloning : pSM2600$^{NotI/XmaI}$+pSM2600$^{osm7499\text{-}osm7205}$	Single integration at *ura4*
pSM2825	pUra4^{PmeI}-p^{nmt1}-fus1N$^{140\text{-}792}$-sfGFP	Infusion cloning : pSM2600$^{NotI/XmaI}$+pSM2600$^{osm7638\text{-}osm7205}$	Single integration at *ura4*
pSM2827	pFA6a-fus1$^{5'UTR}$-fus1-sfGFP-kanMX-fus1$^{3'UTR}$	Infusion cloning : pSM1538$^{SalI/EcoRV}$+IBC180$^{osm7119\text{-}osm5452}$	Single integration at endogenous *fus1* locus
pSM2912	pFA6a-fus1$^{5'UTR}$-fus1$^{\Delta 492\text{-}500}$-sfGFP-kanMX-fus1$^{3'UTR}$	Infusion cloning : pSM2507$^{AfeI/PacI}$+pSM2507$^{osm7127\text{-}osm7738}$ +pSM2507$^{osm7739\text{-}osm7122}$	Single integration at endogenous *fus1* locus
pSM2913	pFA6a-fus1$^{5'UTR}$-fus1-kanMX-fus1$^{3'UTR}$	Infusion cloning : pSM2827$^{EcoRI/AscI}$+pSM2827$^{osm7690\text{-}osm7740}$	Single integration at endogenous *fus1* locus
pSM2937	pFA6a-fus1$^{5'UTR}$-fus1$^{1\text{-}491}$-CRY2PHR-fus1$^{792\text{-}1372}$-sfGFP-kanMX-fus1$^{3'UTR}$	Infusion cloning : pSM2625$^{SalI/XhoI}$+pSM2625$^{osm7119\text{-}osm7878}$ +pSM2475$^{osm7879\text{-}osm7880}$+pSM2625$^{osm7881\text{-}osm7882}$	Single integration at endogenous *fus1* locus
pSM2938	pFA6a-fus1$^{5'UTR}$-fus1$^{1\text{-}491}$-CRY2olig-fus1$^{792\text{-}1372}$-sfGFP-kanMX-fus1$^{3'UTR}$	Infusion cloning : pSM2625$^{SalI/XhoI}$+pSM2625$^{osm7119\text{-}osm7878}$ +pSM2390$^{osm7879\text{-}osm7880}$+pSM2625$^{osm7881\text{-}osm7882}$	Single integration at endogenous *fus1* locus
pSM2939	pFA6a-fus1$^{5'UTR}$-fus1$^{\Delta 731\text{-}791}$-sfGFP-kanMX-fus1$^{3'UTR}$	Infusion cloning : pSM2827$^{XhoI/NheI}$+pSM2827$^{osm7677\text{-}osm7875}$ +pSM2827$^{osm7876\text{-}osm7877}$	Single integration at endogenous *fus1* locus
pSM2940	pFA6a-fus1$^{5'UTR}$-fus1$^{1\text{-}491}$-FUS-fus1$^{792\text{-}1372}$-sfGFP-kanMX-fus1$^{3'UTR}$	Infusion cloning : pSM2625$^{XhoI/SwaI}$+fus1$^{XhoIsite\text{-}491}$-FUS-fus1$^{792\text{-}SwaIsite}$ ordered as a gBlock	Single integration at endogenous *fus1* locus
pSM2941	pFA6a-fus1$^{5'UTR}$-fus1$^{1\text{-}491}$-FUS^{12E}-fus1$^{792\text{-}1372}$-sfGFP-kanMX-fus1$^{3'UTR}$	Infusion cloning : pSM2625$^{XhoI/SwaI}$+fus1$^{XhoIsite\text{-}491}$-FUS^{12E}-fus1$^{792\text{-}SwaIsite}$ ordered as a gBlock	Single integration at endogenous *fus1* locus
pSM3032	pFA6a-fus1$^{5'UTR}$-fus1$^{1\text{-}491}$-FUS^{G156E}-fus1$^{792\text{-}1372}$-sfGFP-kanMX-fus1$^{3'UTR}$	Infusion cloning : pSM2827$^{XhoI/NheI}$+pSM2940$^{osm7677\text{-}osm8388}$ +pSM2940$^{osm8389\text{-}osm7877}$	Single integration at endogenous *fus1* locus
pSM3034	pFA6a-fus1$^{5'UTR}$-fus1$^{1\text{-}491}$-CRY2PHR-fus1$^{792\text{-}1372}$-kanMX-fus1$^{3'UTR}$	Subcloning : pSM2913$^{SalI/NheI}$+pSM2937$^{NotI/XmaI}$	Single integration at endogenous *fus1* locus
pSM3035	pFA6a-fus1$^{5'UTR}$-fus1$^{1\text{-}491}$-CRY2olig-fus1$^{792\text{-}1372}$-kanMX-fus1$^{3'UTR}$	Subcloning : pSM2913$^{SalI/NheI}$+pSM2938$^{NotI/XmaI}$	Single integration at endogenous *fus1* locus
pSM3036	pFA6a-fus1$^{5'UTR}$-fus1$^{1\text{-}491}$-FUS-fus1$^{792\text{-}1372}$-kanMX-fus1$^{3'UTR}$	Subcloning : pSM2913$^{SalI/NheI}$+pSM2940$^{NotI/XmaI}$	Single integration at endogenous *fus1* locus
pSM3037	pFA6a-fus1$^{5'UTR}$-fus1$^{1\text{-}491}$-FUS^{12E}-fus1$^{792\text{-}1372}$-kanMX-fus1$^{3'UTR}$	Subcloning : pSM2913$^{SalI/NheI}$+pSM2941$^{NotI/XmaI}$	Single integration at endogenous *fus1* locus
pSM3055	pUra4^{PmeI}-p^{nmt1}-fus1-mCherry	Infusion cloning : pSM2602$^{XmaI/SacI}$+pSM684$^{osm8480\text{-}osm8481}$ +pSM2602$^{osm8482\text{-}osm8483}$	Single integration at *ura4*
pSM3056	pUra4^{PmeI}-p^{nmt1}-fus1-mCherry	Infusion cloning : pSM2600$^{XmaI/SacI}$+pSM684$^{osm8484\text{-}osm8481}$ +pSM2602$^{osm8482\text{-}osm8483}$	Single integration at *ura4*
