# Supplementary material for: Condensation of the fusion focus by the intrinsically disordered region of the formin Fus1 is essential for cell-cell fusion
Source: Curr Biol. 2022 Nov 7;32(21):4752–4761.e10. doi: 10.1016/j.cub.2022.09.026 (PMC9671092; doi:10.1016/j.cub.2022.09.026)
Supplement: Document S2. Article plus supplemental information [file mmc4.pdf]

Report

Current Biology

Condensation of the fusion focus by the intrinsically disordered region of the formin Fus1 is essential for cell-cell fusion

Graphical abstract

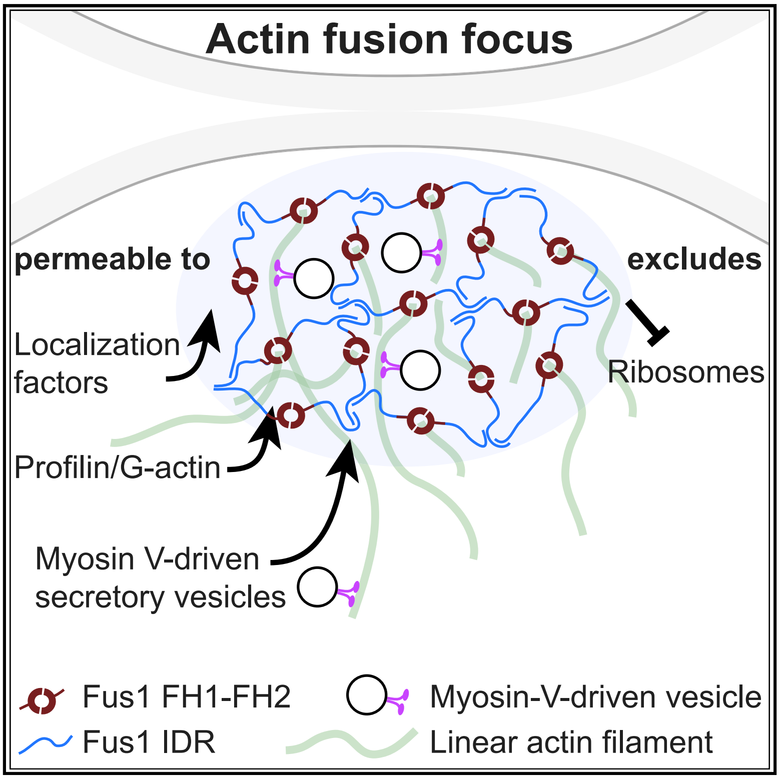

Authors

Ingrid Billault-Chaumartin, Olivia Muriel, Laetitia Michon, Sophie G. Martin

Correspondence

sophie.martin@unil.ch

In brief

Different formin-family nucleators assemble actin networks with distinct architectures. Billault-Chaumartin et al. show that the condensation of the actin focus, which concentrates secretory vesicles for yeast gamete fusion, requires an intrinsically disordered region in the formin Fus1, which can be replaced by heterologous condensate-forming domains.

Highlights

- Fus1 intrinsically disordered region (IDR) promotes assemblies excluding ribosomes
- Fus1 IDR is essential for fusion focus condensation and precise cell wall digestion
- Fus1 IDR can be functionally replaced by FUS^{LC}, which forms molecular condensates
- Fus1 condensation is tuned to yield a permeable focus

Billault-Chaumartin et al., 2022, Current Biology *32*, 4752–4761
November 7, 2022 © 2022 The Author(s). Published by Elsevier Inc.
https://doi.org/10.1016/j.cub.2022.09.026

CellPress

CellPress
OPEN ACCESS

Current Biology

Report

Condensation of the fusion focus by the intrinsically disordered region of the formin Fus1 is essential for cell-cell fusion

Ingrid Billault-Chaumartin,[1] Olivia Muriel,[1] Laetitia Michon,[1] and Sophie G. Martin[1,2,*]
[1]Department of Fundamental Microbiology, Faculty of Biology and Medicine, University of Lausanne, Biophore Building, 1015 Lausanne, Switzerland
[2]Lead contact
*Correspondence: sophie.martin@unil.ch
https://doi.org/10.1016/j.cub.2022.09.026

SUMMARY

Secretory vesicle clusters transported on actin filaments by myosin V motors for local secretion underlie various cellular processes, such as neurotransmitter release at neuronal synapses,[1] hyphal steering in filamentous fungi,[2,3] and local cell wall digestion preceding the fusion of yeast gametes.[4] During fission yeast *Schizosaccharomyces pombe* gamete fusion, the actin fusion focus assembled by the formin Fus1 concentrates secretory vesicles carrying cell wall digestive enzymes.[5–7] The position and coalescence of the vesicle focus are controlled by local signaling and actin-binding proteins to prevent inappropriate cell wall digestion that would cause lysis,[6,8–10] but the mechanisms of focusing have been elusive. Here, we show that the regulatory N terminus of Fus1 contains an intrinsically disordered region (IDR) that mediates Fus1 condensation *in vivo* and forms dense assemblies that exclude ribosomes. Fus1 lacking its IDR fails to concentrate in a tight focus and causes cell lysis during attempted cell fusion. Remarkably, the replacement of Fus1 IDR with a heterologous low-complexity region that forms molecular condensates fully restores Fus1 focusing and function. By contrast, the replacement of Fus1 IDR with a domain that forms more stable oligomers restores focusing but poorly supports cell fusion, suggesting that condensation is tuned to yield a selectively permeable structure. We propose that condensation of actin structures by an IDR may be a general mechanism for actin network organization and the selective local concentration of secretory vesicles.

RESULTS AND DISCUSSION

Formins form a large family of linear F-actin nucleation factors, whose actin-assembly properties are conferred by the formin-homology 1 (FH1) and FH2 domains and regulated by their large, divergent N-terminal region. During fission yeast sexual reproduction, the formin Fus1 assembles the actin fusion focus, which serves to concentrate secretory vesicles transported by the myosin V Myo52 for local cell wall digestion between gametes. Fus1 has actin-assembly properties tailored to its function and cannot be replaced by either of the other two fission yeast formins, For3 and Cdc12.[11] We found that replacement of just Fus1 N terminus (Fus1N) with For3 or Cdc12 N terminus also did not support cell fusion (Figures 1A–1C). Cdc12N-Fus1C failed to localize. For3N-Fus1C localized, like Fus1, to the cell-cell contact region, albeit over a wider zone (Figure 1B). Thus, Fus1N is essential for function, likely by regulating localization and another property.

Fus1N has localization and self-association properties

We first studied Fus1N in interphase cells in which endogenous Fus1 is not expressed. Full-length Fus1, expressed as control, formed a prominent focus rich in linear F-actin (Figure S1A). This focus recruited Myo52 and localized preferentially at one cell pole, which was thinner, or at the division site, occasionally leading to cell lysis after division (Figure 1D; Video S1). F-actin depolymerization by latrunculin A did not affect focus formation but displaced Myo52 (Figure 1D). Thus, Fus1 is active when expressed in mitotic cells and, as during sexual reproduction, likely concentrates secretion leading to cell thinning and lysis. Different from mating cells, Fus1 formed additional clusters that appeared inactive and did not recruit Myo52.

Fus1N (aa 1–792) contains a GBD/FH3 domain that mediates localization,[15] followed by an intrinsically disordered region (IDR), as predicted by tools such as ODiNPred,[12] IUPred3,[13] and PONDR[14] (Figure 1F). In the Alphafold2[16,17] prediction, this IDR is not entirely unstructured but has a few alpha-helices. When expressed in interphase cells, Fus1N exhibited a dual localization to cell tips and cytosolic clusters (Figures 1E and 1G). The cell tip localization overlapped with Myo52, but clusters did not colocalize with Myo52 or linear F-actin and were not perturbed by F-actin depolymerization, consistent with Fus1N lacking actin-assembly domains (Figures 1E, S1A, and S1B). Shortening Fus1N from the N terminus led to the progressive loss of cell tip localization (Fus1N$^{93–792}$, Fus1N$^{140–792}$, and Fus1N$^{191–792}$; Figures 1F–1G). C-terminal truncation of Fus1N IDR led to a loss of cytosolic clusters (Fus1N$^{1–730}$ and Fus1N$^{1–500}$; Figures 1F–1G). When shortened from both ends,

 © 2022 The Author(s). Published by Elsevier Inc.
This is an open access article under the CC BY license (http://creativecommons.org/licenses/by/4.0/).

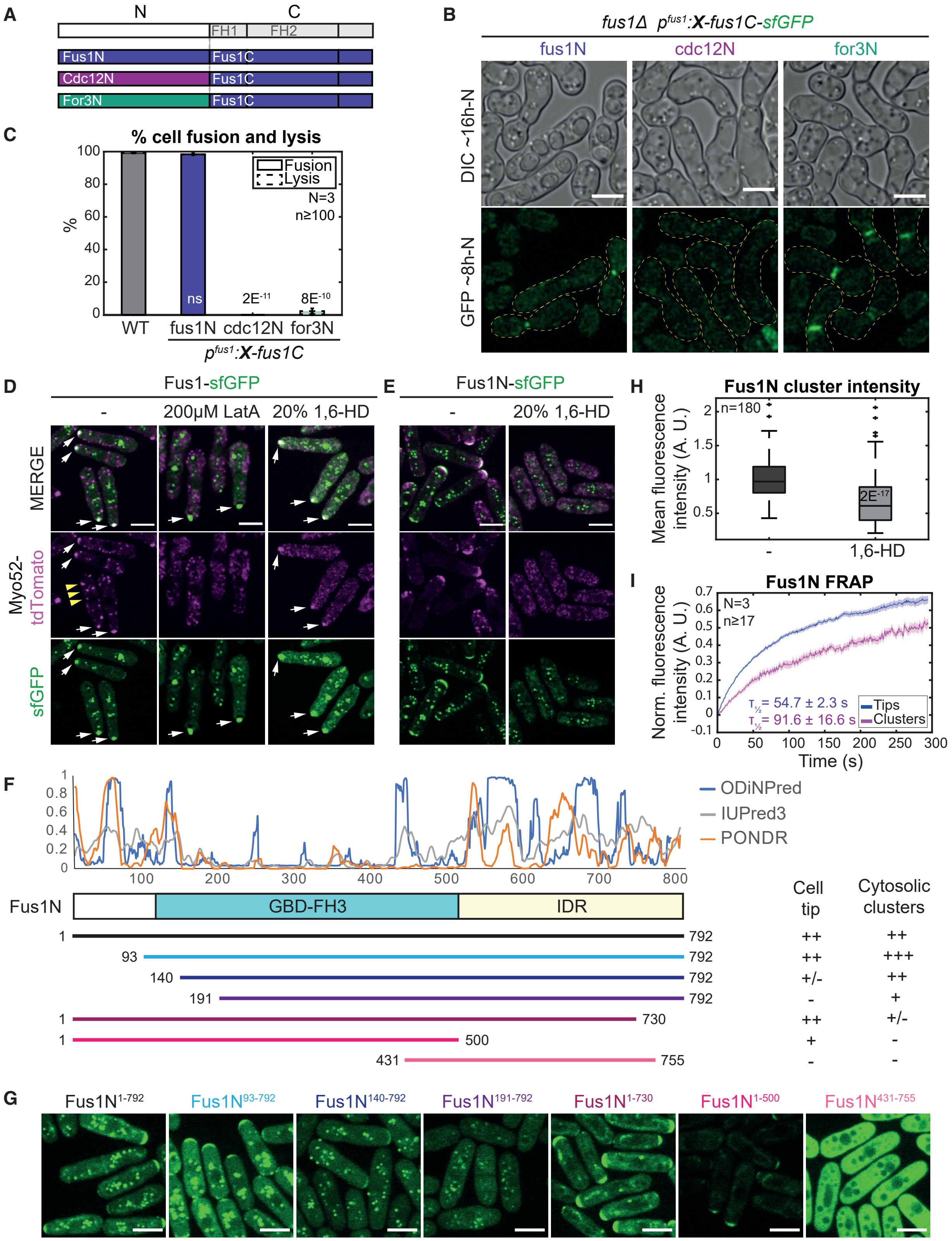

Figure 1. Fus1N is essential for fusion and has localization and self-association properties

(A) Formin chimeras tagged C-terminally with sfGFP.

(B) DIC and GFP images ∼16 and ∼8 h post starvation of *fus1Δ* cells expressing the chimeric formins shown in (A). Yellow dashed lines outline mating pairs.

(legend continued on next page)

Fus1N lost both localizations (Fus1N$^{431–755}$; Figures 1F–1G). Thus, at least in mitotic cells, the Fus1 N-terminal extremity contains localization determinants, while the IDR is necessary for cluster formation.

Fus1N expression modified cellular growth patterns. Wild-type (WT) cells normally grow in a bipolar manner and localize CRIB-labeled Cdc42-GTP, actin assembly, Myo52, and the microtubule-transported Tea1 marker to both cell poles.[18,19] By contrast, Fus1N$^{1–792}$-expressing cells often showed Cdc42-GTP, linear F-actin, and Myo52 at one pole and Tea1 at the other (Figures 1E, S1A, and S1D), like monopolar *tea4Δ* mutants.[20] Fus1N$^{1–792}$ itself localized at the CRIB-labeled cell pole. Fus1N$^{1–730}$, which retains localization but not clustering determinants, induced monopolarity more potently, but Fus1$^{93–792}$ in which localization is compromised did not (Figure S1E). This suggests that Fus1N binding at the cell tip interferes with polarity factors, preventing growth initiation at the second cell pole.

To probe the nature of the Fus1N clusters, we exposed them to high temperature or 1,6-hexanediol, treatments that compromise weak interactions[21] and severely disturbed the localization of Myo52 tagged in the same cell. The high temperature did not affect Fus1N localization (Figure S1C). Treatment with 20% 1,6-hexanediol, an aliphatic alcohol that interferes with hydrophobic interactions and is widely used for disrupting liquid-liquid phase separated (LLPS) condensates,[22] dissipated the cell tip localization of Fus1N and reduced Fus1N clusters, although they were still present, suggesting a solid core (Figures 1E and 1H). Fluorescence recovery after photobleaching (FRAP) experiments further suggested higher stability of Fus1N in cytosolic clusters than at cell tips: only about 50% of the Fus1N cluster signal was mobile and recovered more slowly than the larger mobile pool at cell poles (Figure 1I). High temperature and 1,6-hexanediol also did not disrupt the cytosolic clusters or the larger focus of Fus1 full length to which Myo52 remained associated (Figure 1D, white arrows). Thus, the recruitment of Myo52 upon actin polymerization by Fus1 may trap the motor protein (and likely associated vesicles) in the Fus1 structure. Taken together, these experiments indicate that Fus1N forms resistant assemblies in mitotic cells.

Fus1 foci are zones of ribosome exclusion

In correlative light electron microscopy (CLEM) studies, we previously reported that fusion foci accumulate secretory vesicles but exclude ribosomes and other organelles,[7] suggesting they represent membrane-less organelles. We confirmed this finding by acquiring CLEM-tomograms of Fus1-sfGFP labeled cell pairs lacking the capping protein β subunit Acp2. In the absence of capping proteins, Fus1 is present not only at the fusion focus but is also active on actin patches, where it diverts Myo52 and secretory vesicles, leading to their reduction at the fusion focus.[23] Indeed, the ultrastructure of the fusion site in *acp2Δ* showed a large region devoid of ribosomes with reduced density of secretory vesicles (Figures 2A–2D), indicating local macromolecular exclusion by molecular crowding and/or actin assembly independently of the presence of secretory vesicles.

In mitotic cells, the CLEM of the bright Fus1-sfGFP signal also revealed large regions of ribosome exclusion, whose density was less homogeneous than during mating. In agreement with Myo52 recruitment by Fus1, in 15 of 19 tomograms, vesicles were found in close proximity. However, vesicles were less abundant, smaller, less dense, and more peripheral than at the fusion focus during cell mating (compare Figures 2A, 2E, and 2F). To test whether Fus1 can promote macromolecular exclusion independently of actin assembly, we further acquired CLEM-tomograms of Fus1N, which lacks actin-assembly capacity. We chose Fus1N$^{93–792}$ because this fragment forms prominent cytosolic clusters. In 26 of 30 tomograms, the Fus1N$^{93–792}$-sfGFP fluorescence signal was positioned within 100 nm (corresponding to the precision of the correlation) of a 100–300 nm-wide cytosolic region devoid of ribosomes (Figures 2G–2H). In 23 of these, the region was also darker than the surrounding cytosol. Together, these experiments in mitotic cells show that, independently of actin assembly, Fus1 IDR underlies the formation of large structures that exclude macromolecules such as ribosomes.

Fus1 IDR concentrates Fus1 and is essential for fusion

Fus1N (expressed under the *fus1* or *nmt1* promoter) localized to the contact region between mating cells, as previously reported,[15] but its precise distribution was different in *fus1Δ* and WT cells. When expressed in addition to endogenous WT Fus1, Fus1N localized to the fusion focus marked by Myo52 (Figures 3A and 3D). In *fus1Δ*, Fus1N decorated the entire cell-cell contact area, with measurements along the plasma membrane showing a nearly 2-fold broader distribution (Figures 3A–3D), indicating that Fus1N associates with the fusion focus.

Fus1N$^{191–792}$, which lacks localization information in mitotic cells (see Figures 1F–1G), also failed to localize to the contact site of mating *fus1Δ* cells but was still recruited to the fusion focus in WT cells (Figure 3D), indicating this fragment lost localization determinants but retained fusion focus association. Conversely, Fus1N$^{1–730}$ and Fus1N$^{1–500}$, which fail to form

(C) Percentage of cell pair fusion and lysis 24 h post starvation in WT and strains as in (B). p values relative to WT.

(D) Interphase cells expressing Myo52-tdTomato and full-length Fus1-sfGFP from the *nmt1* promotor. Cells were either untreated (left), treated with 200 μM latrunculin A (middle), or with 20% 1,6-hexanediol (right) for 5 min. White arrows mark resistant fusion focus-like structure; yellow arrowheads indicate labile Myo52 dots.

(E) Interphase cells expressing Myo52-tdTomato and Fus1N-sfGFP (Fus1$^{1–792}$) from the *nmt1* promoter. Cells were either untreated (left) or treated with 20% 1,6-hexanediol for 5 min (right).

(F) Scheme of Fus1N with predicted domain organization. The top graph shows the disorder index of 3 prediction tools.[12–14] Fragments were C-terminally tagged with sfGFP. The localization summary is shown on the right.

(G) GFP-fluorescence images of constructs as in (F).

(H) Boxplot of Fus1 clusters mean fluorescence intensity of cells as in (E). The p value relative to untreated condition.

(I) Average Fus1N FRAP recovery curves normalized to pre-bleach values in cells as in (E). The mean recovery half-time and standard deviation are indicated. N = 3 independent experiments, with n > 17 cells each (n > 54 cells in total). The shaded area shows the standard error. Scale bars, 5 μm.

See also Figure S1.

CellPress
OPEN ACCESS

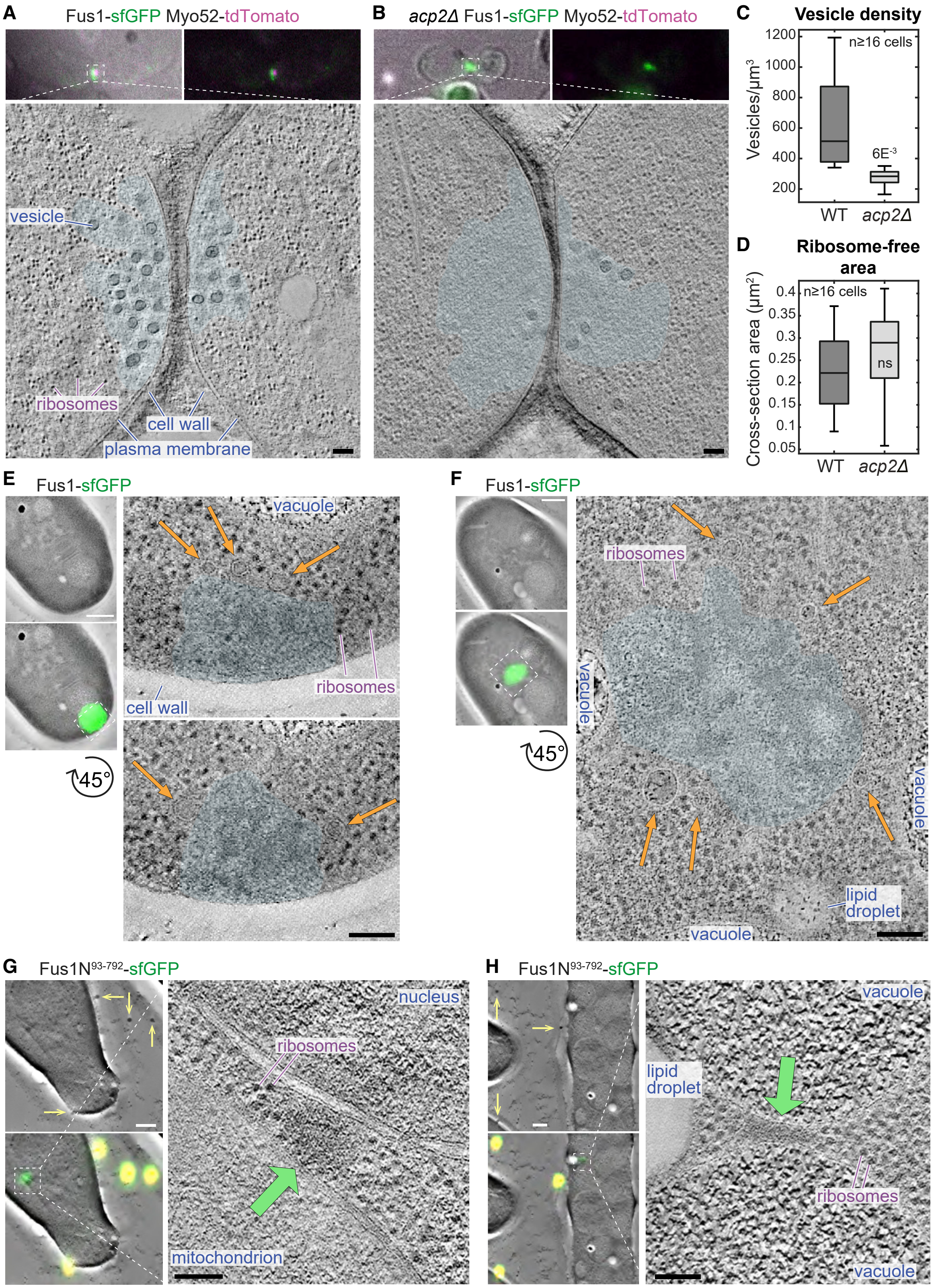

(legend on next page)

cytosolic clusters, localized over a broad region at the fusion site even in WT cells (Figures 3D–3E). This indicates cluster formation and fusion focus association both depend on the IDR, likely through self-interaction.

To test the functional relevance of Fus1 IDR, we deleted it (aa 492–791) from full-length Fus1 expressed from the endogenous locus. Fus1$^{\Delta IDR}$ localized correctly to the site of cell-cell contact but over a wider area than WT Fus1 (Figures 3F–3G) and, in FRAP experiments, recovered faster than WT Fus1 (Figures 3H and 4G), consistent with loss of self-interaction. Fus1$^{\Delta IDR}$ did not support cell fusion (Figure 3I). Instead, a large fraction of cell pairs lysed, likely due to reduced spatial precision of cell wall digestion. Thus, Fus1 IDR strongly contributes to the concentration and function of Fus1 in a focus.

We further dissected the IDR by creating three smaller deletions (Figure 3F), Fus1$^{\Delta 492–500}$, Fus1$^{\Delta 501–749}$, and Fus1$^{\Delta 731–791}$. These mutants showed minor or no phenotype (Figure 3I), suggesting that several elements within the IDR act additively. Combining these deletions two-by-two to create Fus1$^{\Delta 492–749}$ and Fus1$^{\Delta 501–791}$ yielded intermediate phenotypes, and all three (Fus1$^{\Delta IDR}$ described above) had the strongest phenotype (Figure 3I). The distribution of the Fus1 variants at the cell-cell contact site largely mirrored their functionality (Figures 3F–3G), with the least functional ones showing the broadest distribution, though we likely lack resolution in our assay to distinguish some of the intermediate distributions. These observations agree with the idea that the Fus1 IDR condenses the fusion focus through multivalent interactions to promote precise cell wall digestion for cell fusion.

Fus1 IDR can be functionally replaced by heterologous self-assembling domains

If Fus1 IDR mediates multivalent self-interactions, we hypothesized it may be functionally swapped with heterologous domains known to self-assemble (Figure 4A). We first used CRY2^{PHR}, the light-sensitive domain from *Arabidopsis* CRYPTOCHROME 2,[24] which oligomerizes upon blue light exposure[25–27] and can promote LLPS.[28,29] Oligomerization is exacerbated in the CRY2^{olig} mutant.[30] Remarkably, the addition of CRY2^{PHR} or CRY2^{olig} to Fus1$^{\Delta IDR}$ produced formin proteins that were fusion-incompetent in the dark, like the *fus1$^{\Delta IDR}$* mutant, but formed a focus and partly supported cell fusion in the light (Figures 4A–4C). Thus, self-interaction through a heterologous domain can yield a functional fusion focus.

However, several observations distinguished the Fus1$^{\Delta IDR}$-CRY2 fusion foci from the WT, which may explain the partial functionality of the constructs. First, in pairs that successfully fused, foci of CRY2 variants showed a higher local concentration at fusion time than the WT (Figure 4D). Second, in cell pairs that failed to fuse, foci initially positioned at the cell-cell contact detached and moved away in each partner cell (Figure 4A; Video S2). Third, FRAP experiments showed that the CRY2^{PHR} and CRY2^{olig} variants recovered substantially more slowly than the WT (Figure 4G). As these phenotypes are exacerbated by the CRY2^{olig} variants, these observations suggest that self-interaction through CRY2 instead of Fus1 IDR confers excessive focus aggregation, which may in turn impede the entry of other proteins. Indeed, Fus1$^{\Delta IDR}$-CRY2 foci exhibited very low levels of linear F-actin (labeled with mNG-Cdc8[31]) and Myo52, which likely explains their partial functionality (Figures 4H and 4I). We note that the CRY2 addition may also alter other aspects of Fus1 function, as these constructs suppressed the lysis phenotype of *fus1$^{\Delta IDR}$* mutant in the dark (Figure 4C).

With the aim to create a more fluid focus, we swapped Fus1 IDR with the low-complexity domain of the mammalian fused-in-sarcoma protein (FUS^{LC}). FUS^{LC} forms liquid condensates *in vivo* and *in vitro*, which age into solid fibrillar hydrogels.[32–36] We also used a phosphomimetic version, FUS^{12E}, shown to reduce aggregation and form more liquid structures.[37] Strikingly, the replacement of Fus1 IDR by FUS^{LC} or FUS^{12E} produced a fully functional formin that formed a concentrated focus at the fusion site, assembled linear F-actin, recruited Myo52, and supported cell-cell fusion to WT levels and with normal kinetics (Figures 4A, 4B, 4E, and 4F). Local amounts of linear F-actin and Myo52 were indistinguishable or very close to those observed in WT (Figures 4H and 4I). IDR-FUS swaps also showed FRAP half-times much closer to, albeit a bit longer than, WT Fus1 (Figure 4G). The IDR-FUS^{LC} swap did not exhibit gain-of-function in focus formation, as focalization remained dependent on type V myosins, as previously shown[6] (Figure S2). Thus, the condensation properties of Fus1 IDR can be functionally fully replaced by a heterologous self-assembling domain, which confirms the function of Fus1 IDR in self-assembly. It also demonstrates that this region fulfills no other essential function.

Finally, we swapped Fus1 IDR with FUS^{G156E}, a FUS^{LC} variant recently shown to reduce dynamics and promote gelation.[38] Although cell pairs eventually fused successfully (Figure 4E), the kinetics of fusion was significantly slower (Figure 4F). We hypothesize that the condensation properties of Fus1 may be tuned to yield a functional, selectively permeable fusion focus.

The gradual loss of clustering and function upon the progressive deletion of Fus1 IDR suggests it supports weak, multivalent interactions, similar to those exhibited by the FUS^{LC} domain. The strength of these interactions may drive a fluid condensation permeable to actin-assembly factors such as actin-profilin and to myosin-driven cargoes, forming a cluster of secretory vesicles. Fus1 condensation to high local concentration provides an explanation for why key mutations in the FH2 domain that abolish actin assembly *in vitro* (at lower concentrations) only

Figure 2. Fus1 assemblies exclude ribosomes

(A and B) Virtual z-slices through electron tomograms taken at the contact site of (A) WT and (B) *acp2Δ* cell pairs during the fusion process. The transparent cyan shape outlines regions devoid of ribosomes. Images on top show the transmitted light image and fluorescence of Fus1-sfGFP (green) and Myo52-tdTomato (magenta).

(C) Vesicle density at the contact zone. The p value is shown.

(D) Cross-section area of the ribosome-free zone in cells as in (A) and (B).

(E–H) Virtual z-slices through electron tomograms of vegetative cells at the position of Fus1-sfGFP (E and F) or Fus1N$^{93–792}$-sfGFP (G and H). Images on the left show tomograms with and without the correlated fluorescence image (green) and fiducial beads (yellow and arrows in G and H).

Scale bars, 100 nm, except for (E)–(H) (left), 500 nm. See also Video S1.

CellPress
OPEN ACCESS

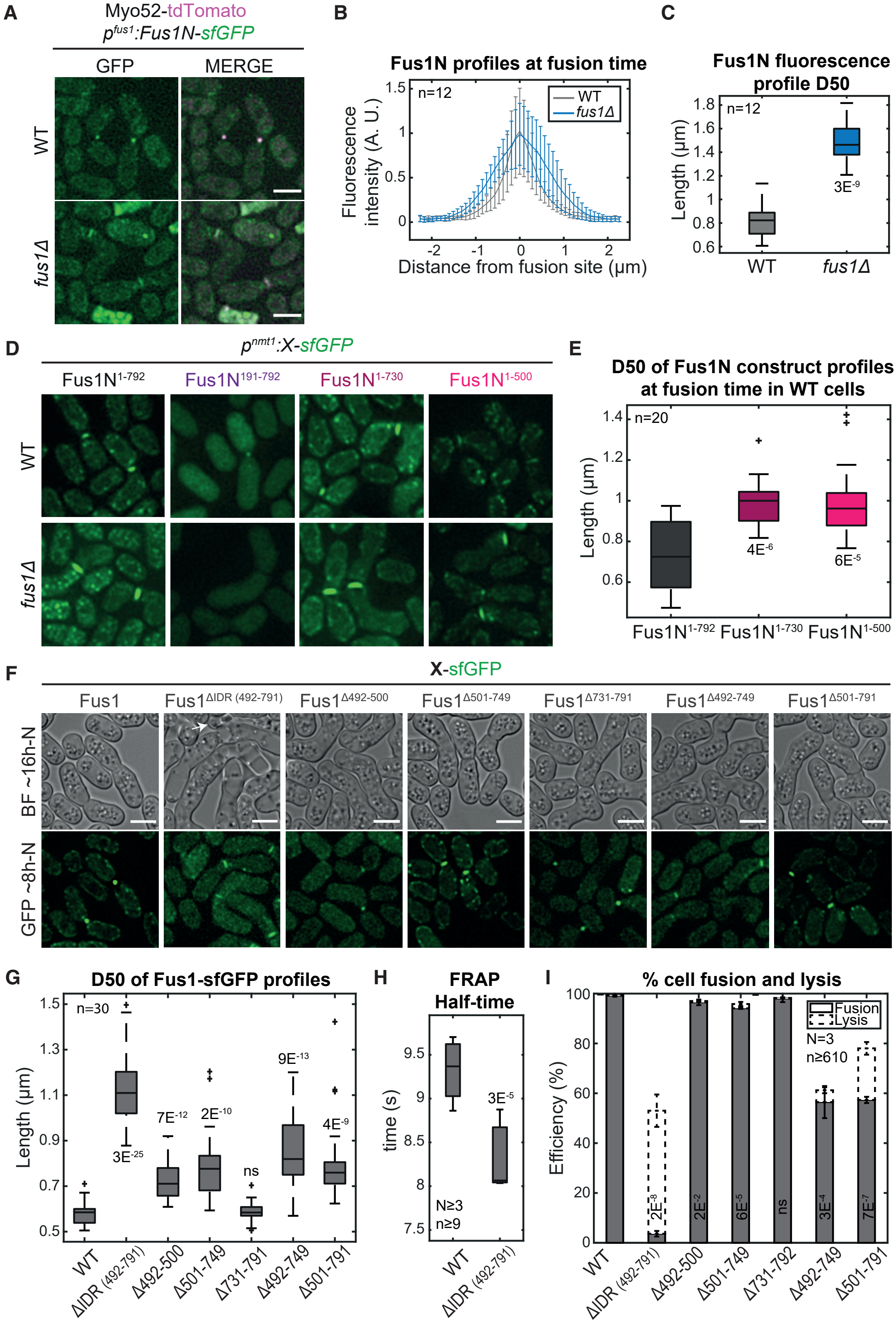

(legend on next page)

partly compromise fusion focus assembly.[23,39] Self-interactions may need to be weak to achieve a balance between condensation and binding to polarity factors, for the correct location of the focus. By contrast, a stronger aggregation that solidifies the structure, as in the CRY2 constructs, likely restricts permeability and access, leading to detachment and lack of function. The apparent fluidity of the fusion focus contrasts with the solid clusters of Fus1N in mitotic cells. This may be due to the regulated accumulation of Fus1 upon sexual differentiation. Alternatively, with pheromone-MAPK signaling present at the fusion focus,[8] Fus1 condensation properties may be regulated by potential post-translational modifications during sexual differentiation.

The condensation properties of Fus1 formin necessary to yield a focus that concentrates secretory vesicles for local cell wall digestion are reminiscent of the role of the synapsin protein, which phase separates to organize clusters of synaptic vesicles at neuronal synapses.[40] Synapsin also bundles and promotes the assembly of actin filaments.[41] A similar mechanism may take place in budding yeast, and likely other fungi, where the formin-binding polarisome factor Spa2 was recently shown to phase separate,[42] likely promoting formin-dependent actin assembly to concentrate secretory vesicles at growth sites. Biomolecular condensation by scaffolds linking to linear actin filaments, or in fission yeast directly by the formin nucleating the structure, may be a general principle by which to organize the focusing of secretory vesicles.

STAR★METHODS

Detailed methods are provided in the online version of this paper and include the following:

- KEY RESOURCES TABLE
- RESOURCE AVAILABILITY
  - Lead contact
  - Materials availability
  - Data and code availability
- EXPERIMENTAL MODEL AND SUBJECT DETAILS
- METHOD DETAILS
  - Strain construction
  - Growth Conditions prior imaging
  - Microscopy
- QUANTIFICATION AND STATISTICAL ANALYSIS

SUPPLEMENTAL INFORMATION

Supplemental information can be found online at https://doi.org/10.1016/j.cub.2022.09.026.

ACKNOWLEDGMENTS

We thank Mohan Balasubramanian for the strains and Aleksander Vjestica, Boris Sieber, Sjoerd Seekles, Sajjita Saha, and Alejandro Melero-Carrillo for the careful reading of the manuscript. This work was funded by grants from the Swiss National Science Foundation (310030B_176396 and 310030_191990) and the European Research Council (CoG CellFusion).

AUTHOR CONTRIBUTIONS

I.B.-C. and S.G.M. conceived the project. S.G.M. performed the experiments in Figure 3A. O.M. performed the experiments in Figure 2. I.B.-C. performed all other experiments with technical assistance from L.M. S.G.M. acquired funding and coordinated the project. I.B.-C. and S.G.M. wrote the first draft of the manuscript, which was revised by all authors.

DECLARATION OF INTERESTS

The authors declare no competing interests.

Received: May 6, 2022
Revised: August 18, 2022
Accepted: September 13, 2022
Published: October 5, 2022

REFERENCES

1. Reshetniak, S., and Rizzoli, S.O. (2021). The vesicle cluster as a major organizer of synaptic composition in the short-term and long-term. Curr. Opin. Cell Biol. *71*, 63–68. https://doi.org/10.1016/j.ceb.2021.02.007.
2. Riquelme, M., and Sánchez-León, E. (2014). The Spitzenkörper: a choreographer of fungal growth and morphogenesis. Curr. Opin. Microbiol. *20*, 27–33. https://doi.org/10.1016/j.mib.2014.04.003.
3. Roberson, R.W. (2020). Subcellular structure and behaviour in fungal hyphae. J. Microsc. *280*, 75–85. https://doi.org/10.1111/jmi.12945.
4. Sieber, B., Coronas-Serna, J.M., and Martin, S.G. (2022). A focus on yeast mating: from pheromone signaling to cell-cell fusion. Semin. Cell Dev. Biol. https://doi.org/10.1016/j.semcdb.2022.02.003.
5. Petersen, J., Weilguny, D., Egel, R., and Nielsen, O. (1995). Characterization of fus1 of *Schizosaccharomyces pombe*: a developmentally controlled function needed for conjugation. Mol. Cell. Biol. *15*, 3697–3707. https://doi.org/10.1128/MCB.15.7.3697.
6. Dudin, O., Bendezú, F.O., Groux, R., Laroche, T., Seitz, A., and Martin, S.G. (2015). A formin-nucleated actin aster concentrates cell wall hydrolases for cell fusion in fission yeast. J. Cell Biol. *208*, 897–911. https://doi.org/10.1083/jcb.201411124.
7. Muriel, O., Michon, L., Kukulski, W., and Martin, S.G. (2021). Ultrastructural plasma membrane asymmetries in tension and curvature promote yeast cell fusion. J. Cell Biol. *220*, e202103142. https://doi.org/10.1083/jcb.202103142.
8. Dudin, O., Merlini, L., and Martin, S.G. (2016). Spatial focalization of pheromone/MAPK signaling triggers commitment to cell-cell fusion. Genes Dev. *30*, 2226–2239. https://doi.org/10.1101/gad.286922.116.

Figure 3. Fus1 IDR concentrates Fus1 and is essential for fusion
(A) Merge and GFP images ~8 h post starvation of Myo52-tdTomato and Fus1N-sfGFP (Fus1$^{1-792}$) expressed in *fus1Δ* or WT cells.
(B) Normalized Fus1N-sfGFP fluorescence profiles perpendicular to the mating pair axis at the time of cell fusion, in strains as in (A).
(C) Width at half-maximum (D50) of the profiles shown in (B).
(D) Fluorescence images ~8 h post starvation of *fus1Δ* or WT cells expressing the indicated Fus1N-sfGFP allele.
(E) D50 of GFP-fluorescence profiles in strains as in (D), in the WT background.
(F) DIC and fluorescence images ~16 and ~8 h post starvation of cells expressing the indicated Fus1-sfGFP allele from the native *fus1* locus. The white arrow points to a lysed pair. Lysis is under-represented, as it mostly happens at later time points.
(G) D50 of GFP-fluorescence profiles in strains as in (F).
(H) Boxplot of WT and *fus1^{ΔIDR}* FRAP half-times.
(I) Percentage of cell pair fusion and lysis 24 h post starvation in strains as in (F). p values relative to left-most strain. Scale bars, 5μm.

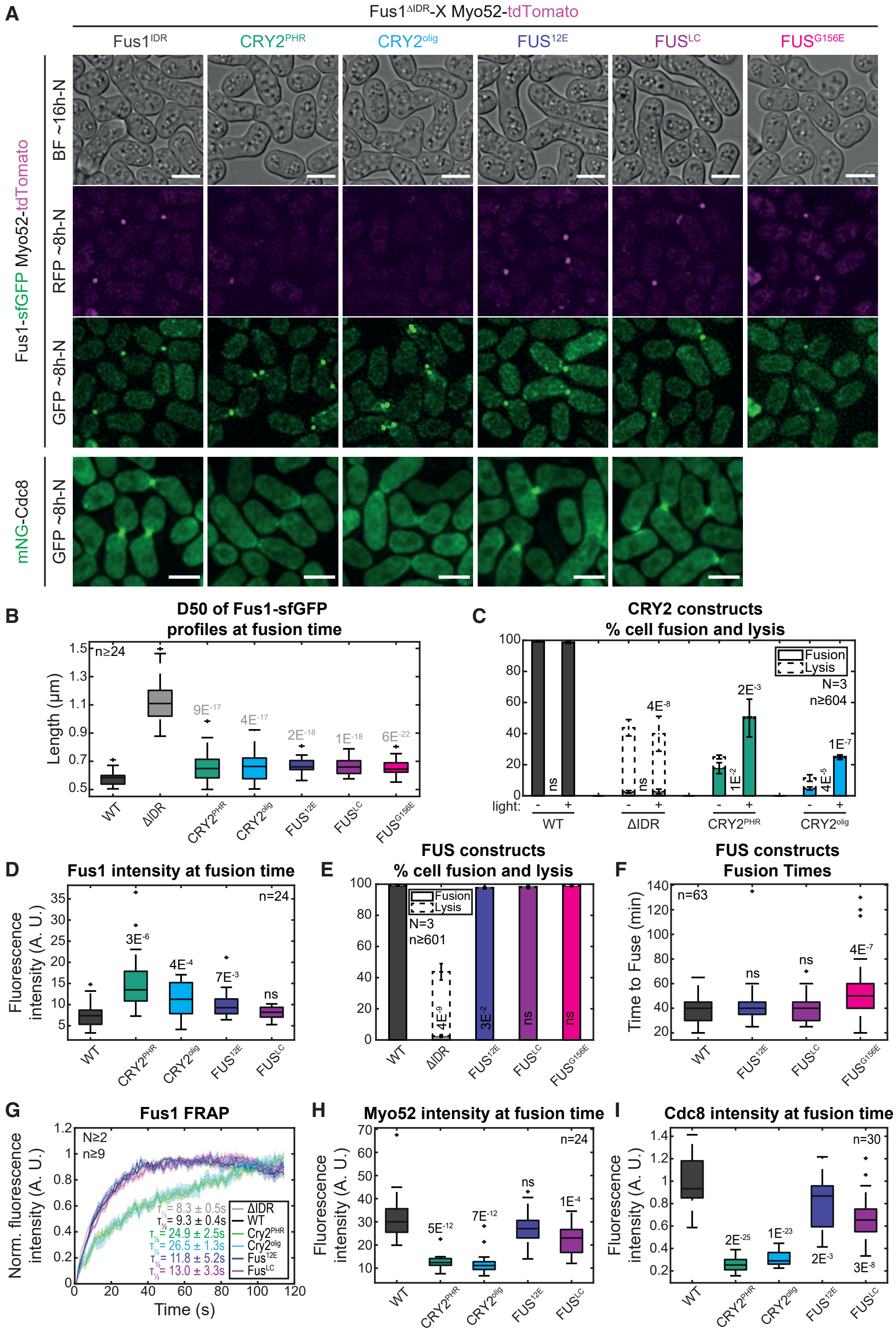

(legend on next page)

9. Merlini, L., Khalili, B., Dudin, O., Michon, L., Vincenzetti, V., and Martin, S.G. (2018). Inhibition of Ras activity coordinates cell fusion with cell-cell contact during yeast mating. J. Cell Biol. *217*, 1467–1483. https://doi.org/10.1083/jcb.201708195.

10. Dudin, O., Merlini, L., Bendezú, F.O., Groux, R., Vincenzetti, V., and Martin, S.G. (2017). A systematic screen for morphological abnormalities during fission yeast sexual reproduction identifies a mechanism of actin aster formation for cell fusion. PLoS Genet. *13*, e1006721. https://doi.org/10.1371/journal.pgen.1006721.

11. Billault-Chaumartin, I., Michon, L., Anderson, C.A., Yde, S.E., Suarez, C., Iwaszkiewicz, J., Zoete, V., Kovar, D.R., and Martin, S.G. (2022). The actin assembly requirements of the formin Fus1 to build the fusion focus. J. Cell Sci. *135*, jcs260289. https://doi.org/10.1101/2022.03.24.485616.

12. Dass, R., Mulder, F.A.A., and Nielsen, J.T. (2020). ODiNPred: comprehensive prediction of protein order and disorder. Sci. Rep. *10*, 14780. https://doi.org/10.1038/s41598-020-71716-1.

13. Erdős, G., Pajkos, M., and Dosztányi, Z. (2021). IUPred3: prediction of protein disorder enhanced with unambiguous experimental annotation and visualization of evolutionary conservation. Nucleic Acids Res. *49*, W297–W303. https://doi.org/10.1093/nar/gkab408.

14. Romero, P., Obradovic, Z., Li, X., Garner, E.C., Brown, C.J., and Dunker, A.K. (2001). Sequence complexity of disordered protein. Proteins *42*, 38–48. https://doi.org/10.1002/1097-0134(20010101)42:1<38::aid-prot50>3.0.co;2-3.

15. Petersen, J., Nielsen, O., Egel, R., and Hagan, I.M. (1998). FH3, a domain found in formins, targets the fission yeast formin Fus1 to the projection tip during conjugation. J. Cell Biol. *141*, 1217–1228. https://doi.org/10.1083/jcb.141.5.1217.

16. Jumper, J., Evans, R., Pritzel, A., Green, T., Figurnov, M., Ronneberger, O., Tunyasuvunakool, K., Bates, R., Žídek, A., Potapenko, A., et al. (2021). Highly accurate protein structure prediction with AlphaFold. Nature *596*, 583–589. https://doi.org/10.1038/s41586-021-03819-2.

17. Varadi, M., Anyango, S., Deshpande, M., Nair, S., Natassia, C., Yordanova, G., Yuan, D., Stroe, O., Wood, G., Laydon, A., et al. (2022). AlphaFold Protein Structure Database: massively expanding the structural coverage of protein-sequence space with high-accuracy models. Nucleic Acids Res. *50*, D439–D444. https://doi.org/10.1093/nar/gkab1061.

18. Tatebe, H., Nakano, K., Maximo, R., and Shiozaki, K. (2008). Pom1 DYRK regulates localization of the Rga4 GAP to ensure bipolar activation of Cdc42 in fission yeast. Curr. Biol. *18*, 322–330. https://doi.org/10.1016/j.cub.2008.02.005.

19. Mata, J., and Nurse, P. (1997). Tea1 and the microtubular cytoskeleton are important for generating global spatial order within the fission yeast cell. Cell *89*, 939–949. https://doi.org/10.1016/s0092-8674(00)80279-2.

20. Martin, S.G., McDonald, W.H., Yates, J.R., 3rd, and Chang, F. (2005). Tea4p links microtubule plus ends with the formin for3p in the establishment of cell polarity. Dev. Cell *8*, 479–491. https://doi.org/10.1016/j.devcel.2005.02.008.

21. Alberti, S., Gladfelter, A., and Mittag, T. (2019). Considerations and challenges in studying liquid-liquid phase separation and biomolecular condensates. Cell *176*, 419–434. https://doi.org/10.1016/j.cell.2018.12.035.

22. Shi, M., You, K., Chen, T., Hou, C., Liang, Z., Liu, M., Wang, J., Wei, T., Qin, J., Chen, Y., et al. (2021). Quantifying the phase separation property of chromatin-associated proteins under physiological conditions using an anti-1,6-hexanediol index. Genome Biol. *22*, 229. https://doi.org/10.1186/s13059-021-02456-2.

23. Billault-Chaumartin, I., and Martin, S.G. (2019). Capping protein insulates Arp2/3-assembled actin patches from formins. Curr. Biol. *29*, 3165–3176.e6. https://doi.org/10.1016/j.cub.2019.07.088.

24. Kennedy, M.J., Hughes, R.M., Peteya, L.A., Schwartz, J.W., Ehlers, M.D., and Tucker, C.L. (2010). Rapid blue-light-mediated induction of protein interactions in living cells. Nat. Methods *7*, 973–975. https://doi.org/10.1038/nmeth.1524.

25. Lamas, I., Merlini, L., Vještica, A., Vincenzetti, V., and Martin, S.G. (2020). Optogenetics reveals Cdc42 local activation by scaffold-mediated positive feedback and Ras GTPase. PLoS Biol. *18*, e3000600. https://doi.org/10.1371/journal.pbio.3000600.

26. Gerganova, V., Lamas, I., Rutkowski, D.M., Vještica, A., Castro, D.G., Vincenzetti, V., Vavylonis, D., and Martin, S.G. (2021). Cell patterning by secretion-induced plasma membrane flows. Sci. Adv. *7*, eabg6718. https://doi.org/10.1126/sciadv.abg6718.

27. Bugaj, L.J., Choksi, A.T., Mesuda, C.K., Kane, R.S., and Schaffer, D.V. (2013). Optogenetic protein clustering and signaling activation in mammalian cells. Nat. Methods *10*, 249–252. https://doi.org/10.1038/nmeth.2360.

28. Wang, X., Jiang, B., Gu, L., Chen, Y., Mora, M., Zhu, M., Noory, E., Wang, Q., and Lin, C. (2021). A photoregulatory mechanism of the circadian clock in Arabidopsis. Nat. Plants *7*, 1397–1408. https://doi.org/10.1038/s41477-021-01002-z.

29. Shin, Y., Berry, J., Pannucci, N., Haataja, M.P., Toettcher, J.E., and Brangwynne, C.P. (2017). Spatiotemporal control of intracellular phase transitions using light-activated optoDroplets. Cell *168*, 159–171.e14. https://doi.org/10.1016/j.cell.2016.11.054.

30. Taslimi, A., Vrana, J.D., Chen, D., Borinskaya, S., Mayer, B.J., Kennedy, M.J., and Tucker, C.L. (2014). An optimized optogenetic clustering tool for probing protein interaction and function. Nat. Commun. *5*, 4925. https://doi.org/10.1038/ncomms5925.

31. Hatano, T., Lim, T.C., Billault-Chaumartin, I., Dhar, A., Gu, Y., Massam-Wu, T., Scott, W., Adishesha, S., Chapa-y-Lazo, B., Springall, L., et al. (2022). mNeonGreen-tagged fusion proteins and nanobodies reveal localization of tropomyosin to patches, cables, and contractile actomyosin rings in live yeast cells. Preprint at bioRxiv. https://doi.org/10.1101/2022.05.19.492673.

32. Patel, A., Lee, H.O., Jawerth, L., Maharana, S., Jahnel, M., Hein, M.Y., Stoynov, S., Mahamid, J., Saha, S., Franzmann, T.M., et al. (2015). A liquid-to-solid phase transition of the ALS protein FUS accelerated by

Figure 4. Fus1 IDR can be functionally replaced by self-assembling domains

(A) DIC and fluorescence images ∼16 and ∼8 h post starvation of cells expressing the indicated Fus1 allele from the native *fus1* locus either tagged with sfGFP and in combination with Myo52-tdTomato (upper panels) or untagged and in combination with mNeonGreen-Cdc8. FUS and CRY2 variants were introduced in Fus1$^{\Delta492–791}$. Cells were exposed to blue light every 5 min for several hours.

(B) Width at half-maximum (D50) of Fus1-sfGFP-fluorescence profiles in strains as in (A). *fus1*$^{\Delta492–791}$ is shown for comparison.

(C) Percentage of cell pair fusion and lysis 24 h post starvation under continuous white light (+) or in the dark (−) in strains with Fus1 or Fus1-CRY2 alleles.

(D) Boxplot of Fus1-sfGFP focus fluorescence intensity at fusion time.

(E) Percentage of cell pair fusion and lysis 24 h post starvation in strains with Fus1 or Fus1-FUS alleles.

(F) Boxplot of fusion times in strains with Fus1 or Fus1-FUS alleles.

(G) Average Fus1 FRAP recovery curves normalized to the maximal recovery value. The mean recovery half-time and the standard deviation are indicated. N = 4, 3, and 2 experiments for the WT, *fus1*$^{\Delta492–791}$, and the 4 other alleles, respectively, with n > 9 cells each (n > 47 cells in total). The shaded area shows the standard error.

(H) Boxplot of Myo52 focus fluorescence intensity at fusion time in strains as indicated.

(I) Boxplot of Cdc8 focus fluorescence intensity at fusion time in strains as indicated. Scale bars, 5 μm. Black p values aligned with bars are relative to WT; gray ones to *fus1*$^{\Delta492–791}$; p values between bars compare the two conditions.

See also Figure S2 and Video S2.

disease mutation. Cell *162*, 1066–1077. https://doi.org/10.1016/j.cell.2015.07.047.

33. Murakami, T., Qamar, S., Lin, J.Q., Schierle, G.S., Rees, E., Miyashita, A., Costa, A.R., Dodd, R.B., Chan, F.T., Michel, C.H., et al. (2015). ALS/FTD mutation-induced phase transition of FUS liquid droplets and reversible hydrogels into irreversible hydrogels impairs RNP granule function. Neuron *88*, 678–690. https://doi.org/10.1016/j.neuron.2015.10.030.

34. Kato, M., Han, T.W., Xie, S., Shi, K., Du, X., Wu, L.C., Mirzaei, H., Goldsmith, E.J., Longgood, J., Pei, J., et al. (2012). Cell-free formation of RNA granules: low complexity sequence domains form dynamic fibers within hydrogels. Cell *149*, 753–767. https://doi.org/10.1016/j.cell.2012.04.017.

35. Sun, Z., Diaz, Z., Fang, X., Hart, M.P., Chesi, A., Shorter, J., and Gitler, A.D. (2011). Molecular determinants and genetic modifiers of aggregation and toxicity for the ALS disease protein FUS/TLS. PLoS Biol. *9*, e1000614. https://doi.org/10.1371/journal.pbio.1000614.

36. Burke, K.A., Janke, A.M., Rhine, C.L., and Fawzi, N.L. (2015). Residue-by-residue view of in vitro FUS granules that bind the C-terminal domain of RNA poly-merase II. Mol. Cell *60*, 231–241. https://doi.org/10.1016/j.molcel.2015.09.006.

37. Monahan, Z., Ryan, V.H., Janke, A.M., Burke, K.A., Rhoads, S.N., Zerze, G.H., O'Meally, R., Dignon, G.L., Conicella, A.E., Zheng, W., et al. (2017). Phosphorylation of the FUS low-complexity domain disrupts phase separation, aggregation, and toxicity. EMBO J. *36*, 2951–2967. https://doi.org/10.15252/embj.201696394.

38. Rhine, K., Makurath, M.A., Liu, J., Skanchy, S., Lopez, C., Catalan, K.F., Ma, Y., Fare, C.M., Shorter, J., Ha, T., et al. (2020). ALS/FTLD-linked mutations in FUS glycine residues cause accelerated gelation and reduced interactions with wild-type FUS. Mol. Cell *80*, 666–681.e8. https://doi.org/10.1016/j.molcel.2020.10.014.

39. Scott, B.J., Neidt, E.M., and Kovar, D.R. (2011). The functionally distinct fission yeast formins have specific actin-assembly properties. Mol. Biol. Cell *22*, 3826–3839. https://doi.org/10.1091/mbc.E11-06-0492.

40. Milovanovic, D., Wu, Y., Bian, X., and De Camilli, P. (2018). A liquid phase of synapsin and lipid vesicles. Science *361*, 604–607. https://doi.org/10.1126/science.aat5671.

41. Cesca, F., Baldelli, P., Valtorta, F., and Benfenati, F. (2010). The synapsins: key actors of synapse function and plasticity. Prog. Neurobiol. *91*, 313–348. https://doi.org/10.1016/j.pneurobio.2010.04.006.

42. Xie, Y., Sun, J., Han, X., Turšić-Wunder, A., Toh, J.D.W., Hong, W., Gao, Y.G., and Miao, Y. (2019). Polarisome scaffolder Spa2-mediated macromolecular condensation of Aip5 for actin polymerization. Nat. Commun. *10*, 5078. https://doi.org/10.1038/s41467-019-13125-1.

43. Vještica, A., Marek, M., Nkosi, P.J., Merlini, L., Liu, G., Bérard, M., Billault-Chaumartin, I., and Martin, S.G. (2020). A toolbox of stable integration vectors in the fission yeast Schizosaccharomyces pombe. J. Cell Sci. *133*, jcs240754. https://doi.org/10.1242/jcs.240754.

44. Mastronarde, D.N. (2005). Automated electron microscope tomography using robust prediction of specimen movements. J. Struct. Biol. *152*, 36–51. https://doi.org/10.1016/j.jsb.2005.07.007.

45. Kremer, J.R., Mastronarde, D.N., and McIntosh, J.R. (1996). Computer visualization of three-dimensional image data using IMOD. J. Struct. Biol. *116*, 71–76. https://doi.org/10.1006/jsbi.1996.0013.

46. Egel, R., Willer, M., Kjaerulff, S., Davey, J., and Nielsen, O. (1994). Assessment of pheromone production and response in fission yeast by a halo test of induced sporulation. Yeast *10*, 1347–1354. https://doi.org/10.1002/yea.320101012.

47. Vjestica, A., Merlini, L., Dudin, O., Bendezu, F.O., and Martin, S.G. (2016). Microscopy of fission yeast sexual lifecycle. J. Vis. Exp. 53801. https://doi.org/10.3791/53801.

48. Bähler, J., Wu, J.Q., Longtine, M.S., Shah, N.G., McKenzie, A., 3rd, Steever, A.B., Wach, A., Philippsen, P., and Pringle, J.R. (1998). Heterologous modules for efficient and versatile PCR-based gene targeting in *Schizosaccharomyces pombe*. Yeast *14*, 943–951. https://doi.org/10.1002/(SICI)1097-0061(199807)14:10<943::AID-YEA292>3.0.CO;2-Y.

49. Kukulski, W., Schorb, M., Welsch, S., Picco, A., Kaksonen, M., and Briggs, J.A. (2012). Precise, correlated fluorescence microscopy and electron tomography of Lowicryl sections using fluorescent fiducial markers. Methods Cell Biol. *111*, 235–257. https://doi.org/10.1016/B978-0-12-416026-2.00013-3.

50. Mastronarde, D.N., and Held, S.R. (2017). Automated tilt series alignment and tomographic reconstruction in IMOD. J. Struct. Biol. *197*, 102–113. https://doi.org/10.1016/j.jsb.2016.07.011.

51. Vicente, N.B., Zamboni, J.E.D., Adur, J.F., Paravani, E.V., and Casco, V.H. (2007). Photobleaching correction in fluorescence microscopy images. J. Phys.: Conf. Ser. *90*, 012068. https://doi.org/10.1088/1742-6596/90/1/012068.

52. Das, M., Drake, T., Wiley, D.J., Buchwald, P., Vavylonis, D., and Verde, F. (2012). Oscillatory dynamics of Cdc42 GTPase in the control of polarized growth. Science *337*, 239–243. https://doi.org/10.1126/science.1218377.

53. Paul-Gilloteaux, P., Heiligenstein, X., Belle, M., Domart, M.C., Larijani, B., Collinson, L., Raposo, G., and Salamero, J. (2017). eC-CLEM: flexible multidimensional registration software for correlative microscopies. Nat. Methods *14*, 102–103. https://doi.org/10.1038/nmeth.4170.

STAR★METHODS

KEY RESOURCES TABLE

REAGENT or RESOURCE	SOURCE	IDENTIFIER
**Chemicals, peptides, and recombinant proteins**		
LatrunculinA	Enzo Life Science	Cat# BML-T-119-0500
AgaPure Agarose LE	Promega	Cat# V3125
Vaselin	Reactolab	Cat# 99813
Lanolin	Fluka	Cat# 49909
Paraffin	Reactolab	Cat# 99756
Dimethyl sulfoxide (DMSO)	Applichem	Cat# A3672
Poly(ethylene glycol) BioUltra, 4,000	Sigma	Cat# 95904
Lithium Acetat Dihydrat	Applichem	Cat# A3478
EDTA Disodium Salt 2-hydrate	Applichem	Cat# A2937
Tris(hydroxymethyl)aminomethane	Biosolve	Cat# 200923
1,6-hexanediol	Sigma	Cat# 240117-506
Lowicryl HM20 Embedding Kit	Electron Microscopy Sciences	Cat# 14340
Reynolds lead citrate	Sigma	467863
Acetone	Sharlau	AC03101000
Uranyl Acetate	Fluka	94260
**Experimental models: Organisms/strains**		
h90 myo52-tdTomato:natMX fus1-sfGFP:kanMX ura4- leu1-32 ade6-M216	Lab Stock[23]	YSM3312
h90 myo52-tdTomato:natMX fus1Δ::LEU2+ ura4-294:p^{fus1}-fus1N^{1}-^{792}-fus1C$^{793-1372}$-sfGFP:ura4+ leu1-32	This work	YSM2504
h90 myo52-tdTomato:natMX fus1Δ::LEU2+ ura4-294:p^{fus1}-cdc12N^{1}-^{887}-fus1C$^{793-1372}$-sfGFP:ura4+ leu1-32	This work	YSM2512
h90 myo52-tdTomato:natMX fus1Δ::LEU2+ ura4-294:p^{fus1}-for3N^{1}-^{714}-fus1C$^{793-1372}$-sfGFP:ura4+ leu1-32	This work	YSM2510
h90 myo52-tdTomato:natMX ura4+:p^{nmt1}: fus1N^{1}-^{792}-sfGFP:term^{nmt} leu1-32 ade6-M210	This work	YSM4002
h90 myo52-tdTomato:natMX ura4+:p^{nmt1}: fus1N^{1}-^{730}-sfGFP:term^{nmt} leu1-32 ade6-M210	This work	YSM4003
h90 myo52-tdTomato:natMX ura4+:p^{nmt1}: fus1N^{1}-^{500}-sfGFP:term^{nmt} leu1-32 ade6-M210	This work	YSM4004
h90 myo52-tdTomato:natMX ura4+:p^{nmt1}: fus1N^{93}-^{792}-sfGFP:term^{nmt} leu1-32 ade6-M210	This work	YSM4005
h90 myo52-tdTomato:natMX ura4+:p^{nmt1}: fus1N^{140}-^{792}-sfGFP:term^{nmt} leu1-32 ade6-M210	This work	YSM4006
h90 myo52-tdTomato:natMX ura4+:p^{nmt1}: fus1N^{191}-^{792}-sfGFP:term^{nmt} leu1-32 ade6-M210	This work	YSM4007
h90 myo52-tdTomato:natMX ura4+:p^{nmt1}: fus1N^{431}-^{755}-sfGFP:term^{nmt} leu1-32 ade6-M210	This work	YSM4008
h90 myo52-tdTomato:natMX ura4+:p^{nmt1}: fus1-sfGFP:term^{nmt} leu1-32 ade6-M210	This work	YSM4009
h90 leu1-32:p^{cdc8}:mNeonGreen-cdc8:term^{cdc8}: term^{ScADH1}:leu1+ ura4-D18 ade6-M216	This work	YSM3786
h90 ura4+:p^{nmt1}:fus1-mCherry:term^{nmt} leu1-32:p^{cdc8}: mNeonGreen-cdc8:term^{cdc8}:term^{ScADH1}:leu1+ ade6-M216	This work	YSM4042
h90 ura4+:p^{nmt1}:fus1N^{1}-^{792}-mCherry:term^{nmt} leu1-32:pcdc8: mNeonGreen-cdc8:term^{cdc8}:term^{ScADH1}:leu1+ ade6-M216	This work	YSM4043

(*Continued on next page*)

CellPress
OPEN ACCESS

Continued

REAGENT or RESOURCE	SOURCE	IDENTIFIER
h+ his5+:p^{act1}:CRIB-3mCherry:bsdMX ura4-D18	This work	YSM4010
h90 his5+:p^{act1}:CRIB-3mCherry:bsdMX ura4+: p^{nmt1}:fus1N^{1}-^{792}-sfGFP:term^{nmt} leu1-32	This work	YSM4011
h90 his5+:p^{act1}:CRIB-3mCherry:bsdMX ura4+: p^{nmt1}:fus1N^{1}-^{730}-sfGFP:term^{nmt} ade6-M210	This work	YSM4012
h90 his5+:p^{act1}:CRIB-3mCherry:bsdMX ura4+: p^{nmt1}:fus1N^{93}-^{792}-sfGFP:term^{nmt} leu1-32 ade6-M210	This work	YSM4013
h90 tea1-mCherry:kanMX ura4-D18 leu1-32	This work	YSM4014
h90 tea1-mCherry:kanMX ura4+:p^{nmt1}:fus1N^{1}-^{792}-sfGFP:term^{nmt} leu1-32	This work	YSM4015
h90 tea1-mCherry:kanMX ura4+:p^{nmt1}:fus1N^{1}-^{730}-sfGFP:term^{nmt} leu1-32	This work	YSM4016
h90 tea1-mCherry:kanMX ura4+:p^{nmt1}:fus1N^{93}-^{792}-sfGFP:term^{nmt} leu1-32 ade6-M210	This work	YSM4017
h90 myo52-tdTomato:natMX fus1-sfGFP:kanMX	Lab Stock[7]	YSM3888
h90 myo52-tdTomato:natMX fus1-sfGFP:kanMX acp2Δ::bleMX ura4- leu1-32 ade6-M210	Lab Stock[23]	YSM3314
h90 myo52-tdTomato:natMX ura4+:p^{nmt1}: fus1N^{93}-^{792}-sfGFP:term^{nmt} fus1Δ::hphMX ade6-M210 leu1-32	This work	YSM4018
h90 myo52-tdTomato:natMX ura4+:p^{nmt1}: fus1-sfGFP:term^{nmt} fus1Δ::hphMX ade6-M210 leu1-32	This work	YSM4053
h90 myo52-tdTomato:natMX ura4-294:p^{fus1}: fus1N-sfGFP:ura4+ fus1Δ::LEU2+ leu1-32	This work	YSM2486
h90 myo52-tdTomato:natMX ura4-294:p^{fus1}:fus1N-sfGFP:ura4+ leu1-32	This work	YSM2699
h90 myo52-tdTomato:natMX ura4+:p^{nmt1}: fus1N^{1}-^{792}-sfGFP:term^{nmt} fus1Δ::hphMX leu1-32 ade6-M210	This work	YSM4054
h90 myo52-tdTomato:natMX ura4+:p^{nmt1}: fus1N^{191}-^{792}-sfGFP:term^{nmt} fus1Δ::hphMX leu1-32 ade6-M210	This work	YSM4055
h90 myo52-tdTomato:natMX ura4+:p^{nmt1}: fus1N^{1}-^{730}-sfGFP:term^{nmt} fus1Δ::hphMX leu1-32 ade6-M210	This work	YSM4056
h90 myo52-tdTomato:natMX ura4+:p^{nmt1}: fus1N^{1}-^{500}-sfGFP:term^{nmt} fus1Δ::hphMX leu1-32 ade6-M210	This work	YSM4057
h90 myo52-tdTomato:natMX fus1$^{Δ501\text{-}749}$-sfGFP: kanMX ura4-294 leu1-32 ade6-M210	This work	YSM4019
h90 myo52-tdTomato:natMX fus1$^{Δ501\text{-}791}$-sfGFP: kanMX ura4-294 leu1-32 ade6-M210	This work	YSM4020
h90 myo52-tdTomato:natMX fus1$^{Δ492\text{-}791}$-sfGFP: kanMX ura4-294 leu1-32 ade6-M210	This work	YSM4021
h90 myo52-tdTomato:natMX fus1$^{Δ492\text{-}500}$-sfGFP: kanMX ura4-294 leu1-32 ade6-M210	This work	YSM4044
h90 myo52-tdTomato:natMX fus1$^{Δ731\text{-}791}$-sfGFP: kanMX ura4-294 leu1-32 ade6-M210	This work	YSM4045
h90 myo52-tdTomato:natMX fus1$^{Δ492\text{-}749}$-sfGFP: kanMX ura4-294 leu1-32 ade6-M210	This work	YSM4046
h90 myo52-tdTomato:natMX fus1^{1}-^{491}-FUS^{12E}-fus1$^{792\text{-}1372}$-sfGFP:kanMX ura4-294 leu1-32 ade6-M210	This work	YSM4022
h90 myo52-tdTomato:natMX fus1^{1}-^{491}-FUS-fus1$^{792\text{-}1372}$-sfGFP: kanMX ura4-294 leu1-32 ade6-M210	This work	YSM4023
h90 myo52-tdTomato:natMX fus1^{1}-^{491}-CRY2^{PHR}-fus1$^{792\text{-}1372}$-sfGFP:kanMX ura4-294 leu1-32 ade6-M210	This work	YSM4024
h90 myo52-tdTomato:natMX fus1^{1}-^{491}-CRY2^{olig}-fus1$^{792\text{-}1372}$-sfGFP:kanMX ura4-294 leu1-32 ade6-M210	This work	YSM4025
h90 myo52-tdTomato:natMX fus1^{1}-^{491}-FUS^{G156E}-fus1$^{792\text{-}1372}$-sfGFP:kanMX ura4-294 leu1-32 ade6-M210	This work	YSM4047
h90 myo52-tdTomato:natMX leu1-32:p^{cdc8}:mNeonGreen-cdc8:term^{cdc8}:term^{ScADH1}:leu1+ fus1:kanMX ura4-294 ade6-M210	This work	YSM4026

(*Continued on next page*)

Continued

REAGENT or RESOURCE	SOURCE	IDENTIFIER
h90 myo52-tdTomato:natMX leu1-32:p^{cdc8}:mNeonGreen-cdc8:term^{cdc8}:term^{ScADH1}:leu1+ fus1$^{1-491}$-CRY2^{PHR}-fus1$^{792-1372}$:kanMX ura4-294 ade6-M210	This work	YSM4048
h90 myo52-tdTomato:natMX leu1-32:p^{cdc8}:mNeonGreen-cdc8:term^{cdc8}:term^{ScADH1}:leu1+ fus1$^{1-491}$-CRY2^{olig}-fus1$^{792-1372}$:kanMX ura4-294 ade6-M210	This work	YSM4049
h90 myo52-tdTomato:natMX leu1-32:p^{cdc8}:mNeonGreen-cdc8:term^{cdc8}:term^{ScADH1}:leu1+ fus1$^{1-491}$-FUS-fus1$^{792-1372}$:kanMX ura4-294 ade6-M210	This work	YSM4050
h90 myo52-tdTomato:natMX leu1-32:p^{cdc8}:mNeonGreen-cdc8:term^{cdc8}:term^{ScADH1}:leu1+ fus1$^{1-491}$-FUS^{12E}-fus1$^{792-1372}$:kanMX ura4-294 ade6-M210	This work	YSM4051
h90 fus1-sfGFP:kanMX myo51Δ::ura4+ myo52Δ::ura4+ leu1-32	Lab Stock[6]	YSM2543
h90 fus1$^{1-491}$-FUS-fus1$^{792-1372}$-sfGFP:kanMX myo51Δ::ura4+ myo52Δ::ura4+ leu1-32	This work	YSM4052
Oligonucleotides		
CAGCTCCAAATTTTGAAAGTAAAACCCCTAATTAGGG AATAAATAAGTAGGCAGAGCACCTTGAAAAATAA CTAGATAGAATTCGAGCTCGTTTAAAC	Sigma	osm765
AATAAAAAGAGACAAACAGTCGTCCTTAAAGC TGAATGCATGCTTAAGCAGCTGGAGAATAACAA TGAACTTAAGAGACGGATCCCCGGGTTAATTAA	Sigma	osm932
TTTTATTAATTATAATTTCATTATAATTTGTTTAA GTCATTTAATTGTCATTAAAAGTCATTAACA TTTCAAACATCAGAATTCGAGCTCGTTTAAAC	Sigma	osm933
GATCACTGTAGGCAACGTAGCCGACAATGATGTACA GAACTCGAGCGACGAAGAAAATCAAGTACCAA ATGGTATTAAAGTTCGGATCCCCGGGTTAATTAA	Sigma	osm1196
ACGGATTTCATGAAGTTATTGGTTAAAAGCGGCCT CTCAAATCCTCCAGCTAAAGAACCAGTCCATGAC AACGAAAATCGGATCCCCGGGTTAATTAA	Sigma	osm1746
ATGTCATCGTCGAATATTTACACTATGTACAGTCC TTTCAACTAGTAAAGGAGATGCTTTCAAAATAG TTCCAAAGAGGAATTCGAGCTCGTTTAAAC	Sigma	osm1747
CGTATCACGAGGCCCTTTCG	Sigma	osm1772
CCGGATCCTCCAAGGGTGAAGAGCTATTTACTGGGG	Sigma	osm2217
ACTGCGGCCGCATGATGACGGCTAGTTTTAAAGG	Sigma	osm3005
ACTCCCGGGTCTCTTAAGTTCATTGTTATTCTCC	Sigma	osm3006
ACTGCGGCCGCATGGCATCTAAAATGCCTGAAG	Sigma	osm3007
ACTGCGGCCGCATGCGAAATTCGTCAAAGGGAC	Sigma	osm3009
CTTGGATCCTCATATTTTCTATTTTAGAAAACCTC	Sigma	osm3026
TGAGGATCCAAGAAGTTATTGATGGGAATCC	Sigma	osm3027
CTGGGATCCATGGCGAAGGCGAGGAAG	Sigma	osm3028
TCGGGATCCTACTATTGTTGCTAACTGTTTCTGC	Sigma	osm3030
GTAGGATCCCGAACTTTGATATTCCTAATGATGC	Sigma	osm3031
CGGGGTACCGATCAGAAAATTATCGCCAT	Sigma	osm3091
ACTGCGGCCGCTGATTTAACAAAGCGACTATAAGTC	Sigma	osm3516
ACTCCCGGGAGTAGAAGTGTTAGGAGCTTC	Sigma	osm3521
CTTGGATCCTATGAACCTCAAAAGAATGCGTTG	Sigma	osm4021
CATTAAGGCCTCACTTTTATTCTGAGATCGCTAT CCGGTTGTATTCTTTTGTTTAAAGCATTATATC ATCAACTCACCCGGATCCCCGGGTTAATTAA	Sigma	osm4504

(*Continued on next page*)

CellPress
OPEN ACCESS

Continued

REAGENT or RESOURCE	SOURCE	IDENTIFIER
CAATCTTTCTATGACTATTTTCGTTGAAG<br>ATGGAACGAATACTATGAGAAGAT<br>CACGGAAAGAAAACAAAAAG<br>CAATCGAATTCGAGCTCGTTTAAAC	Sigma	osm4505
GGAATAAGGGCGACACGG	Sigma	osm4577
GGCCACTAGTGGATCTGATA<br>TCGATGTATTTACTGATTACTT	Sigma	osm5452
CTTCTAAACGGCTAGCTCAGCTTCATTGG	Sigma	osm5453
CAATGAAGCTGAGCTAGCCGTTTAGAAGG	Sigma	osm5454
CATATGGTCTGGGTATCT	Sigma	osm6064
GCCTTCCAACCAGCTTCTCT	Sigma	osm6183
CTTGGATCCATCATTATTTGAATTACCAT	Sigma	osm6576
CTTGTTTAAACCAACATGCCTGTAAG	Sigma	osm6582
GAAGTTTAAACTGCTTTTGTGGTTATC	Sigma	osm6583
CTTCGTACGCTGCAGGTCG<br>ACACAGTATGTACGCCAC	Sigma	osm7119
TTCACCCTTGGAGTTAATTA<br>ATCTCTTAAGTTCATTGTTAT	Sigma	osm7122
ATGTACCAGGCGAAGCGCTTCTATGTCCGGATGAC	Sigma	osm7127
CTTCTTTGATTCTCATATCAGCTTGTAAAGTAAGC	Sigma	osm7140
TACTTTACAAGCTGATATGAGAATCAAAGAAGTTAT	Sigma	osm7141
CTTTGTTAAATCAGCGGCCGC<br>ATGTTTACCGATTCATATGTA	Sigma	osm7204
CTTGGAGTTAATTAACCCGGGGATCCTCATATTTTC	Sigma	osm7205
GCTTTGTTAAATCAGCGGCCGCATGATGAC	Sigma	osm7254
CTTGGAGTTAATTAACCCGGG<br>GATCCTATCATTATTTGAATTACCA	Sigma	osm7255
CTTTGTTAAATCAGCGGCC<br>GCATGAAGCACACTCCAAATTCT	Sigma	osm7256
CTTGGAGTTAATTAACCCGGGGATCCTAAA<br>AACCTTGTGTTTTGA	Sigma	osm7257
CTTCTTTGATTCTCATATCATTATTTGAATTACCAT	Sigma	osm7487
TAATTCAAATAATGATATGAGAATCAAAGAAGTTAT	Sigma	osm7488
AAACCTTGTGTTTTGAATCAGCTTGTAAAGTAAG	Sigma	osm7489
TACTTTACAAGCTGATTCAAAACACAAGGTTTTTA	Sigma	osm7490
GCTTTGTTAAATCAGCGGCCGCATGCTCAA<br>GTACGTGGAATCTTT	Sigma	osm7499
CTTTGTTAAATCAGCGGCCGCATGGTTACACTCTCTCAAGAAAA	Sigma	osm7638
GGAGTATTAAAACAACTCGAGAAATGCGTGAAACTC	Sigma	osm7677
AAATCAAGGATATGAGAATTCCGAAAGAAAGTATGT	Sigma	osm7690
TATAAAAGCAATCAATATCAGCTTGTAAAGTAAGCAC	Sigma	osm7738
TACTTTACAAGCTGATATTGATTGCTTTTATAAGGAATTAAAG	Sigma	osm7739
GCTTATTTAGAAGTGGCGCGCCTCTCTTAAGTTCATTGTTATTC	Sigma	osm7740
CTTCTTTGATTCTCATATGAACCTCAAAAGAATGCG	Sigma	osm7875
TTCTTTTGAGGTTCATATGAGAATCAAAGAAGTTATTGAT	Sigma	osm7876
CTTCTGATTTACAGTGCTAGCCTTTTTGTACTCCAGTATTAT	Sigma	osm7877
TTTTGTCCATCTTCATCGTCATCATTAACAAGCAATAG	Sigma	osm7878
CTTGTTAATGATGACGATGAAGATGGACAAAAAGACTAT	Sigma	osm7879
AACTAGCCGTCATCATTGCTGCTCCGATCATGATCT	Sigma	osm7880
CATGATCGGAGCAGCAATGATGACGGCTAGTTTTAAAG	Sigma	osm7881
GAGTTTCACGCATTTCTCGAGTTGTTTTAATACTCCTTC	Sigma	osm7882

(*Continued on next page*)

Continued

REAGENT or RESOURCE	SOURCE	IDENTIFIER
ACTGGTTCTGCTGTTCATAGCCCTGAGGGGGATTA	Sigma	osm8388
CCCTCAGGGCTATGAACAGCAGAACCAGTACAAC	Sigma	osm8389
ACTTAAGAGAGGATCCCCGGGTTAATTAAC	Sigma	osm8480
ATTCCTTTTACCCGGTTTACTTGTACAGCTCGTCC	Sigma	osm8481
CGAGCTGTACAAGTAAACCGGGTAAAAGGAATGTC	Sigma	osm8482
AGGGAACAAAAGCTGGAGC	Sigma	osm8483
GAAAATATGAGGATCCCCGGGTTAATTAAC	Sigma	osm8484
Recombinant DNA		
pUra4^{AfeI}	Vještica et al.[43]	pAV133
pREP3x	Lab Stock	pSM617
pFA6a-mCherry-kanMX	Lab Stock	pSM677
pFA6a-mCherry-natMX	Lab Stock	pSM684
pFA6a-tdTomato-natMX	Lab Stock	pSM685
pFA6a-bleMX	Lab Stock	pSM694
pFA6a-sfGFP-kanMX	Lab Stock	pSM1538
pRIP-p^{fus1}-sfGFP	Lab Stock	pSM1638
pRIP-p^{fus1}-fus1N-sfGFP	This work	pSM1650
pRIP-p^{fus1}-fus1-sfGFP	This work	pSM1656
pRIP-p^{fus1}-fus1N-fus1C-sfGFP	This work	pSM1659
pRIP-p^{fus1}-for3N-fus1C-sfGFP	This work	pSM1662
pRIP-p^{fus1}-cdc12N-fus1C-sfGFP	This work	pSM1663
pRIP-p^{nmt41}-sfGFP	Lab Stock	pSM1823
pRIP-p^{nmt41}-fus1N-sfGFP	This work	pSM1826
pUra4^{AfeI}-p^{nmt41}-fus1-sfGFP	Lab Stock	pSM2229
pFA6a-fus1$^{5'UTR}$-fus1_K879A-sfGFP-kanMX-fus1$^{3'UTR}$	This work	pSM2251
pRIP-p^{fus1}-CRY2olig-For3N-fus1C-sfGFP	Lab Stock	pSM2390
pUra4^{AfeI}-p^{fus1}-CRY2PHR-fus1C-sfGFP	Lab Stock	pSM2475
pUra4^{PmeI}-p^{nmt41}-fus1-sfGFP	This work	pSM2478
pFA6a-fus1$^{5'UTR}$-fus1$^{\Delta 501-749}$-sfGFP-kanMX-fus1$^{3'UTR}$	This work	pSM2507
pUra4^{PmeI}-p^{nmt1}-fus1N-sfGFP	This work	pSM2600
pUra4^{PmeI}-p^{nmt1}-fus1N$^{1-730}$-sfGFP	This work	pSM2601
pUra4^{PmeI}-p^{nmt1}-fus1-sfGFP	This work	pSM2602
pFA6a-fus1$^{5'UTR}$-fus1$^{\Delta 492-791}$-sfGFP-kanMX-fus1$^{3'UTR}$	This work	pSM2625
pUra4^{PmeI}-p^{nmt1}-fus1N$^{93-792}$-sfGFP	This work	pSM2630
pUra4^{PmeI}-p^{nmt1}-fus1N$^{1-500}$-sfGFP	This work	pSM2644
pUra4^{PmeI}-p^{nmt1}-fus1N$^{431-755}$-sfGFP	This work	pSM2645
pFA6a-fus1$^{5'UTR}$-fus1$^{\Delta 501-791}$-sfGFP-kanMX-fus1$^{3'UTR}$	This work	pSM2697
pFA6a-fus1$^{5'UTR}$-fus1$^{\Delta 492-749}$-sfGFP-kanMX-fus1$^{3'UTR}$	This work	pSM2698
pUra4^{PmeI}-p^{nmt1}-fus1N$^{191-792}$-sfGFP	This work	pSM2703
pUra4^{PmeI}-p^{nmt1}-fus1N$^{140-792}$-sfGFP	This work	pSM2825
pFA6a-fus1$^{5'UTR}$-fus1-sfGFP-kanMX-fus1$^{3'UTR}$	This work	pSM2827
pFA6a-fus1$^{5'UTR}$-fus1$^{\Delta 492-500}$-sfGFP-kanMX-fus1$^{3'UTR}$	This work	pSM2912
pFA6a-fus1$^{5'UTR}$-fus1-kanMX-fus1$^{3'UTR}$	This work	pSM2913
pFA6a-fus1$^{5'UTR}$-fus1$^{1-491}$-CRY2PHR-fus1$^{792-1372}$-sfGFP-kanMX-fus1$^{3'UTR}$	This work	pSM2937
pFA6a-fus1$^{5'UTR}$-fus1$^{1-491}$-CRY2olig-fus1$^{792-1372}$-sfGFP-kanMX-fus1$^{3'UTR}$	This work	pSM2938
pFA6a-fus1$^{5'UTR}$-fus1$^{\Delta 731-791}$-sfGFP-kanMX-fus1$^{3'UTR}$	This work	pSM2939
pFA6a-fus1$^{5'UTR}$-fus1$^{1-491}$-FUS-fus1$^{792-1372}$-sfGFP-kanMX-fus1$^{3'UTR}$	This work	pSM2940

(*Continued on next page*)

Continued

REAGENT or RESOURCE	SOURCE	IDENTIFIER
pFA6a-fus1$^{5'UTR}$-fus1$^{1-491}$-FUS^{12E}-fus1$^{792-1372}$-sfGFP-kanMX-fus1$^{3'UTR}$	This work	pSM2941
pFA6a-fus1$^{5'UTR}$-fus1$^{1-491}$-FUS^{G156E}-fus1$^{792-1372}$-sfGFP-kanMX-fus1$^{3'UTR}$	This work	pSM3032
pFA6a-fus1$^{5'UTR}$-fus1$^{1-491}$-CRY2PHR-fus1$^{792-1372}$-kanMX-fus1$^{3'UTR}$	This work	pSM3034
pFA6a-fus1$^{5'UTR}$-fus1$^{1-491}$-CRY2olig-fus1$^{792-1372}$-kanMX-fus1$^{3'UTR}$	This work	pSM3035
pFA6a-fus1$^{5'UTR}$-fus1$^{1-491}$-FUS-fus1$^{792-1372}$-kanMX-fus1$^{3'UTR}$	This work	pSM3036
pFA6a-fus1$^{5'UTR}$-fus1$^{1-491}$-FUS^{12E}-fus1$^{792-1372}$-kanMX-fus1$^{3'UTR}$	This work	pSM3037
pUra4^{PmeI}-p^{nmt1}-fus1-mCherry	This work	pSM3055
pUra4^{PmeI}-p^{nmt1}-fus1-mCherry	This work	pSM3056
gBlock FUSLC: GGAGTATTAAAACAACTCGAGAAATGC<br>GTGAAACTCGTATCATTAGACACTGCTAATGAGAAACA<br>TTTTTTAAAGCACACTCCAAATTCTGCTGCTCATCAATC<br>CCTTTTAAACACAAACATGTTTAATGATGCAAATTTCGA<br>ATTTATGGTTAAAGAGCATATTAAAAATTTTTTAAAACTT<br>TTGAAAGAGCACAACAACCCCGTCCGTATTATAAAGTT<br>ACTTGATTGTTTAGTGCTTACTTTACAAGCTGATATGGC<br>CTCAAACGATTATACCCAACAAGCAACCCAAAGCTATG<br>GGGCCTACCCCACCCAGCCCGGGCAGGGCTATTCCC<br>AGCAGAGCAGTCAGCCCTACGGACAGCAGAGTTACAG<br>TGGTTATAGCCAGTCCACGGACACTTCAGGCTATGGCC<br>AGAGCAGCTATTCTTCTTATGGCCAGAGCCAGAACACA<br>GGCTATGGAACTCAGTCAACTCCCCAGGGATATGGCTC<br>GACTGGCGGCTATGGCAGTAGCCAGAGCTCCCAATCGT<br>CTTACGGGCAGCAGTCCTCCTATCCTGGCTATGGCCAG<br>CAGCCAGCTCCCAGCAGCACCTCGGGAAGTTACGGTAG<br>CAGTTCTCAGAGCAGCAGCTATGGGCAGCCCCAGAGTG<br>GGAGCTACAGCCAGCAGCCTAGCTATGGTGGACAGCAG<br>CAAAGCTATGGACAGCAGCAAAGCTATAATCCCCCTCAG<br>GGCTATGGACAGCAGAACCAGTACAACAGCATGAGAATC<br>AAAGAAGTTATTGATGGGAATCCATTCAAAGCTCCACCTC<br>CTGCACCATTACCACCTCCTGCACCTCCTTTACCAACTGC<br>AATGTCTTCTCTCCAGAAATTTGAAAAAAATGATTCACAAA<br>TTTTTCGGAAGACGATAATTATTCCCGAAAATATTTCAATC<br>GATGACATATTTAAATTCTGTTCAGGTT	Integrated DNA Technologies	REF #: 229090872
gBlock FUS12E: GGAGTATTAAAACAACTCGAGAAATGCGTG<br>AAACTCGTATCATTAGACACTGCTAATGAGAAACATTTTTTAA<br>AGCACACTCCAAATTCTGCTGCTCATCAATCCCTTTTAAACA<br>CAAACATGTTTAATGATGCAAATTTCGAATTTATGGTTAAAGA<br>GCATATTAAAAATTTTTTAAAACTTTTGAAAGAGCACAACAAC<br>CCCGTCCGTATTATAAAGTTACTTGATTGTTTAGTGCTTACTT<br>TACAAGCTGATATGGCCTCAAACGATTATGAGCAACAAGCA<br>GAACAAAGCTATGGGGCCTACCCCGAGCAGCCCGGGCAGG<br>GCTATGAACAGCAGAGCGAGCAGCCCTACGGACAGCAGAG<br>TTACAGTGGTTATGAACAGTCCACGGACACTTCAGGCTATGG<br>CCAGAGCAGCTATTCTTCTTATGGCCAGGAGCAGAACACAG<br>GCTATGGAGAGCAGTCAACTCCCCAGGGATATGGCTCGACT<br>GGCGGCTATGGCAGTGAGCAGAGCGAACAATCGTCTTACGG<br>GCAGCAGTCCTCCTATCCTGGCTATGGCCAGCAGCCAGCTC<br>CCAGCAGCACCTCGGGAAGTTACGGTAGCAGTGAGCAGAG<br>CAGCAGCTATGGGCAGCCCCAGAGTGGGAGCTACGAGCAG<br>CAGCCTAGCTATGGTGGACAGCAGCAAAGCTATGGACAGCA<br>GCAAAGCTATAATCCCCCTCAGGGCTATGGACAGCAGAACC<br>AGTACAACAGCATGAGAATCAAAGAAGTTATTGATGGGAATC<br>CATTCAAAGCTCCACCTCCTGCACCATTACCACCTCCTGCAC<br>CTCCTTTACCAACTGCAATGTCTTCTCTCCAGAAATTTGAAA<br>AAAATGATTCACAAATTTTTCGGAAGACGATAATTATTCCCG<br>AAAATATTTCAATCGATGACATATTTAAATTCTGTTCAGGTT	Integrated DNA Technologies	REF #: 229090873

(*Continued on next page*)

Continued		
REAGENT or RESOURCE	SOURCE	IDENTIFIER
Software and algorithms		
ImageJ (Fiji)	NIH	RRID: SCR_002285
S. pombe database (released in August, 2013)	PomBase	RRID: SCR_006586
Volocity	PerkinElmer	RRID: SCR_002668
softWoRx v4.1.2	Applied Precision, GE Healthcare	No direct download
Matlab	MathWorks	RRID: SCR_001622
ZEN 3.3 (blue edition)	Zeiss	RRID: SCR_013672
SerialEM	Mastronarde [44]	RRID: SCR_017293
IMOD	Kremer et al.[45]	RRID: SCR_003297
AlphaFold2	Varadi et al.[17]	https://alphafold.ebi.ac.uk/
Other		
Fluorescent TetraSpeck beads	Invitrogen	REF: T7279

RESOURCE AVAILABILITY

Lead contact

Further information and requests for resources and reagents should be directed to and will be fulfilled by the Lead Contact, Sophie Martin (sophie.martin@unil.ch).

Materials availability

Plasmids and yeast strains generated in this study have not been deposited on an external repository but are available for distribution on request from the Lead Contact.

Data and code availability

- This study did not generate any substantial dataset. Microscopy data reported in this paper and/or its raw quantification will be shared by the lead contact upon request.
- This study did not generate any substantial code.
- Any additional information required to reanalyze the data reported in this paper is available from the lead contact upon request.

EXPERIMENTAL MODEL AND SUBJECT DETAILS

S. pombe strains used in this study are listed in the key resources table and in Table S1, which links them to each figure. For mating experiments, homothallic (*h90*) strains able to switch mating types were used, where cells were grown in liquid or agar Minimum Sporulation Media (MSL), with or without nitrogen (+/- N).[46,47] For interphase experiments, cells were grown in liquid or agar Edinburgh minimal medium (EMM) supplemented with amino acids as required. In both cases, cells were generally handled at 30°C, unless stated otherwise.

METHOD DETAILS

Strain construction

Strains were constructed using standard genetic manipulation of *S. pombe* either by tetrad dissection or transformation and can be found in the key resources table and their link to figures in Table S1. Plasmids generated for this study are listed in the key resources table, and details on how they were made can be found in Table S2. Oligonucleotides used in this study are listed in the key resources table, and details on their use can be found in Table S3.

myo52-tdTomato:natMX, *fus1-sfGFP:kanMX* and *tea1-mCherry:kanMX* tags were constructed by PCR-based gene targeting[48] of a fragment from a template pFA6a plasmid containing the appropriate tag and resistance cassette, amplified with primers carrying 5' extensions corresponding to the last 78 coding nucleotides of the ORF and the first 78 nucleotides of the 3'UTR, which was transformed and integrated in the genome by homologous recombination, as previously described.[48] Similarly, *acp2Δ::bleMX* was constructed by PCR-based gene targeting of a fragment from a template pFA6a plasmid containing the appropriate resistance cassette, amplified with primers carrying 5' extensions corresponding or the last 78 nucleotides of the 5'UTR and the first 78 nucleotides of the 3'UTR, which was transformed and integrated in the genome by homologous recombination.

Construction of the strains expressing formin constructs from the *fus1* promotor at the *ura4* locus as a multicopy integration (ura4-294:p^{fus1}-fus1N$^{1-792}$-fus1C$^{793-1372}$-sfGFP:ura4+, ura4-294:p^{fus1}-cdc12N$^{1-887}$-fus1C$^{793-1372}$-sfGFP:ura4+, ura4-294:p^{fus1}-for3N$^{1-714}$-fus1C$^{793-1372}$-sfGFP:ura4+, ura4-294:p^{fus1}:fus1N$^{1-792}$-sfGFP:ura4+) was done by homologous recombination of a transformed ura4^{EndORF}-ura4$^{3'UTR}$-p^{fus1}-ForminConstruct-sfGFP-ura4^{StartORF}-ura4$^{5'UTR}$ fragment, obtained from StuI digestion of a pRIP based plasmid (pSM1659, pSM1663, pSM1662 and pSM1650, respectively). Such recombination recreates a new integration site, which has been shown to be unstable and to lead to multiple insertion,[43] which is why we switched to single integration vectors for the rest of the study.

Construction of the strains expressing *fus1* constructs under *nmt1* promotor at the *ura4* locus as a single integration (ura4+:p^{nmt1}:fus1N$^{1-792}$-sfGFP:termnmt, ura4+:p^{nmt1}:fus1N$^{1-730}$-sfGFP:term^{nmt} ura4+:p^{nmt1}:fus1N$^{1-500}$-sfGFP:term^{nmt}, ura4+:p^{nmt1}:fus1N$^{93-792}$-sfGFP:term^{nmt}, ura4+:p^{nmt1}:fus1N$^{140-792}$-sfGFP:term^{nmt}, ura4+:p^{nmt1}:fus1N$^{191-792}$-sfGFP:term^{nmt}, ura4+:p^{nmt1}:fus1N$^{431-755}$-sfGFP:term^{nmt}, ura4+:p^{nmt1}:fus1-sfGFP:term^{nmt}, ura4+:p^{nmt1}:fus1N$^{1-792}$-mCherry:termnmt, ura4+:p^{nmt1}:fus1-sfGFP:termnmt,) was done by homologous recombination of a transformed ura4$^{5'UTR}$-ura4^{ORF}-ura4$^{3'UTR}$-p^{nmt1}-Fus1Construct-sfGFP-ura4$^{3''}$ fragment, obtained from PmeI digestion of a pUra4^{PmeI} based plasmid (pSM2600, pSM2601, pSM2644, pSM2630, pSM2825, pSM2703, pSM2645, pSM2602, pSM3056 and pSM3055, respectively). This leads to a stable single integration at the *ura4* locus.[43]

Construction of the strains expressing formin constructs from the endogenous locus (fus1$^{\Delta 492-500}$-sfGFP:kanMX, fus1$^{\Delta 731-791}$-sfGFP:kanMX, fus1$^{\Delta 492-749}$-sfGFP:kanMX, fus1$^{\Delta 501-749}$-sfGFP:kanMX, fus1$^{\Delta 501-791}$-sfGFP:kanMX, fus1$^{\Delta 492-791}$-sfGFP:kanMX, fus1$^{1-491}$-FUS^{12E}-fus1$^{792-1372}$-sfGFP:kanMX, fus1$^{1-491}$-FUS-fus1$^{792-1372}$-sfGFP:kanMX, fus1$^{1-491}$-FUS^{G156E}-fus1$^{792-1372}$-sfGFP:kanMX, CRY2^{PHR}-fus1$^{1-491}$-fus1$^{792-1372}$-sfGFP:kanMX, CRY2^{olig}-fus1$^{1-491}$-fus1$^{792-1372}$-sfGFP:kanMX, CRY2^{PHR}-fus1$^{1-491}$-fus1$^{792-1372}$:kanMX, CRY2^{olig}-fus1$^{1-491}$-fus1$^{792-1372}$:kanMX, fus1$^{1-491}$-FUS-fus1$^{792-1372}$:kanMX, fus1$^{1-491}$-FUS^{12E}-fus1$^{792-1372}$:kanMX) were done by homologous recombination of a transformed fus1$^{5'UTR}$-ForminConstruct-sfGFP-kanMX-fus1$^{3'UTR}$ fragment, obtained from a gel purified, SalI and SacII digested pFA6a based plasmid (pSM2912, pSM2939, pSM2698, pSM2507, pSM2697, pSM2625, pSM2941, pSM2940, pSM3032, pSM2937, pSM2938, pSM3034, pSM3035, pSM3036 and pSM3037, respectively). FUS fragments are the first 163 amino acids of the human protein. CRY2 fragments are the codon optimized PHR domains from *Arabidopsis thaliana*.

leu1-32:pcdc8:mNeonGreen-cdc8:termcdc8:termScADH1:leu1+,[31] *fus1Δ::LEU2+*[15] and his5+:p^{act1}:CRIB-3mCherry:bsdMX[43] trace back to the aforementioned papers or are kind gifts from the afore mentioned labs.

Growth Conditions prior imaging

Live imaging of *S. pombe* mating cells was adapted from Vjestica et al.[47] Briefly, cells were first pre-cultured overnight in MSL+N at 30°C, then diluted to OD600 = 0.05 into MSL+N at 25°C for 20 hours. Exponentially growing cells were then pelleted, washed in MSL-N by 3 rounds of centrifugation, and resuspended in MSL-N to an OD600 of 1.5. Cells were then grown 3 hours at 30°C to allow mating in liquid, added on 2% agarose MSL-N pads, and sealed with VALAP. We allowed the pads to rest for 30 min at 30°C before overnight imaging, or for 21h at 25°C for fusion efficiencies snapshot imaging, respectively.

For Correlative Light Electron Microscopy (CLEM) imaging, as described in Muriel et al.,[7] cells were grown for mating as described above or at exponential phase as described below. In the case of mating, after washes to remove nitrogen, cells were added into MSL−N plates. We allowed cells to mate for 5 h. A few microliters of MSL−N were pipetted onto the cells to form a thick slurry. In the second case, cells were pelleted by centrifugation. Yeast paste was pipetted onto a 3-mm-wide, 0.1-mm-deep specimen carrier (Wohlwend type A) closed with a flat lid (Wohlwend type B) for high-pressure freezing with a HPM100 (Leica Microsystems; for mating samples) or a Leica EM ICE high-pressure freezer (for interphase cells). The carrier sandwich was disassembled in liquid nitrogen before freeze substitution. High-pressure frozen samples were processed by freeze substitution and embedded in Lowicryl HM20 using the Leica AFS 2 robot as described.[49] 300-nm sections were cut with a diamond knife using a Leica Ultracut E or Ultracut UC7 ultramicrotome, collected in H_2O, and picked up on carbon-coated 200-mesh copper grids (AGS160; Agar Scientific). For Light Microscopy, the grid was inverted onto a 1× PBS drop on a microscope coverslip, which was mounted onto a microscope slide. For Figures 2A and 2B, it was imaged using the DeltaVision platform described below to select for pairs with a Myo52-tdTomato and Fus1-sfGFP signal, indicating presence of a fusion focus. For Figures 2E–2H, fluorescent TetraSpeck beads (Invitrogen), 100 nm in diameter, were adsorbed onto the grid before light microscopy imaging, to be used as fiducials for correlation. The grid was then imaged using the Zeiss LSM980 setup described below, or an epifluorescence microscope Zeiss Axio Imager Z2 using a 63x/1.25 NA oil objective and Hamamatsu ORCA-flash4.0 camera, to capture both Fus1N-sfGFP and fiducial fluorescence signal. The grid was then recovered, rinsed in H_2O, and dried before post-staining with Reynolds lead citrate for 10 min. 15-nm protein A-coupled gold beads were adsorbed to the top of the section as fiducials for tomography.

For interphase imaging, cells were grown to exponential phase at 30°C in EMM+ALU media, pelleted and imaged between slide and coverslip. All strains containing a repressible *nmt* promotor were grown at least 24h without thiamine before imaging to reach maximal expression levels. For 1,6-hexanediol and LatrunculinA treatments in Figures 1D, 1E, and S1B, the drug was added directly before imaging to the final resuspension, to a final concentration of 20% and 200μM, respectively, and cells were imaged right away or after 5 minutes, respectively. For the 37°C treatment in Figure S1C, cells were grown to exponential phase at 30°C in EMM+ALU media, then shifted to 37°C for 6h, transported to the microscope on a 40°C carrier, and imaged at 40°C.

Microscopy

Images presented in Figures 1B, 1D, 1E, 3A, 3D, 3F, 4A, S1A–S1D, and S2A were obtained using a DeltaVision platform (Applied Precision) composed of a customized inverted microscope (IX-71; Olympus), a UPlan Apochromat 100×/1.4 NA oil objective, a

camera (CoolSNAP HQ2; Photometrics or 4.2Mpx PrimeBSI sCMOS camera; Photometrics), and a color combined unit illuminator (Insight SSI 7; Social Science Insights). Images were acquired using softWoRx v4.1.2 software (Applied Precision). Images were acquired every 5 minutes during 9 to 15 hours. To limit photobleaching, overnight videos were captured by optical axis integration (OAI) imaging of a 4.6-μm z-section, which is essentially a real-time z-sweep.

Images presented in Figure 1G were obtained using a spinning-disk microscope composed of an inverted microscope (DMI4000B; Leica) equipped with an HCX Plan Apochromat 100×/1.46 NA oil objective and an UltraVIEW system (PerkinElmer; including a real-time confocal scanning head [CSU22; Yokagawa Electric Corporation], solid-state laser lines, and an electron-multiplying charge coupled device camera [C9100; Hamamatsu Photonics]). Time-lapse images were acquired at 1s interval using the Volocity software (PerkinElmer).

Images used to obtain Figures 1I and 4G were obtained using a ZEISS LSM 980 scanning confocal microscope with 4 confocal Detectors (2x GaAsP, 2x PMT), an Airyscan2 detector optimized for a 60x/1.518 NA oil objective, and 6 Laser Lines (405nm, 445nm, 488nm, 514nm, 561nm, 640nm) on inverted Microscope Axio Observer 7. For Figure 1I we used images acquired using the Airyscan2 detector and processed with the Zen3.3 (blue edition) software for super resolution. For Figure 4G we switched to the confocal mode as the lower fluorescence intensity required. We acquired images every second, and we bleached the cells by 1 iteration of a 25% (1I) or 10% (4G) 488nm laser power pulse after 5 time points and kept recording the fluorescence recovery for 5 (1I) or 2 minutes (4G). Temperature was controlled by an incubation chamber around the microscope.

Images used to obtain Figures 2A, 2B, and 2E–2H were obtained following CLEM, as described in Muriel et al.[7] TEMs were acquired on a FEI Tecnai 12 at 120 kV using a bottom mount FEI Eagle camera (4kx4k). Low-magnification TEM images were acquired at 15.592, 11.39 or 7.63-nm pixel size, low-magnification tomograms at 4.576-nm pixel size and high magnification tomograms at 1.205-nm pixel size. For tomographic reconstruction of regions of interest, one (Figures 2A, 2B, 2G, and 2H) or two (Figures 2E and 2F) axis tilt series were acquired over a tilt range as large as possible up to ±60° at 1° increments using the Serial EM software.[44] The IMOD software package with gold fiducial alignment[45,50] was used for tomogram reconstruction.

QUANTIFICATION AND STATISTICAL ANALYSIS

Percentages of cell fusion and lysis as in Figures 1C, 3H, 4C, and 4E were calculated as in Dudin et al.[6] Briefly, 24h post-starvation, fused cell pairs, lysed pairs and the total number of cell pairs were quantified using the ImageJ Plugin ObjectJ, and percentages were calculated using the following equations:

$$\%\ cell\ fusion = \frac{Fused\ Pairs}{Mating\ Pairs} \times 100$$

$$\%\ cell\ lysis = \frac{Lysed\ Pairs}{Mating\ Pairs} \times 100$$

Fusion Times as in Figure 4F were calculated in overnight time lapse Videos at 5-minutes interval using the 2-dot Myo52-tdTomato stage[6] as a marker for the beginning of the fusion process and either the entry of GFP expressed under control of the P-cell-specific pmap3 promoter into the h- partner, or the maximum intensity of the Myo52-tdTomato dot, the two of which perfectly correlate,[6] as a marker for the end of the process.

Fusion Focus intensities at fusion time as in Figures 3B, 4D, 4H, and 4I were obtained from 5-minutes time lapse overnight Videos using the maximum intensity of the Myo52-tdTomato dot to determine the moment of fusion (which correlates with the entry of GFP expressed under control of the P-cell-specific p^{map3} promoter into the *h-* partner[6]). On that time frame, a fluorescence profile across the fusion focus perpendicular to the long axis of the mating pair was recorded and either used directly as in Figure 3B or only the central point of the profiles were used to obtain boxplots as in Figures 4D, 4H, and 4I. Profiles were background-subtracted and corrected for bleaching as follows: First, the cell fluorescence intensity was recorded over time in a square of about 7x7 pixels in 12 control (non-mating) cell. These fluorescence profiles were averaged, and the mean was fitted to a double exponential as it was describing our data better[51]:

$$Signal_{photobleaching-correction}(t) = Ae^{-Bt} + Ce^{-Dt}$$

We then used this fit to correct the fluorescence profiles across the fusion focus for photobleaching. After subtracting background signal, the value at each timepoint was divided by the photo-bleaching correction signal:

$$Signal_{BleachingCorrected} = \frac{Signal_t - Signal_{Background}}{Signal_{photobleaching-correction}(t)}$$

Corrected profiles were then either directly averaged and plotted (Figures 4D and 4H), or further normalized to the mean of the maximum (Figures 3B and 4I). Widths at half maximum (D50) as in Figures 3C, 3E, 3G, 4B, and S2D were then calculated using these fluorescence profiles by recording (through linear interpolation in between points) the 2 distances that intersected with half maximal intensity for each individual profile, which were subtracted from one another. The result was then plotted as a boxplot.

The monopolar percentage as shown in Figure S1E was assessed from single fluorescence snapshot images of CRIB and classified as monopolar (decorating only one pole) or bipolar (decorating the two poles at similar intensities). The ratio of the first category divided by the sum of the two gave the monopolar percentage. Note that even WT bipolar cells can appear monopolar using this assay, as they can be captured at a time in CRIB oscillations[52] where only one cell tip is decorated.

Clusters intensities as in Figure 1H were calculated from the mean fluorescence intensity of 5 circular ROIs centered on clusters per cell for 36 cells per condition.

The density of vesicles in Figure 2C was obtained by manually counting vesicles within a half cylinder of 1μm diameter centred at the contact site.

The size of the ribosome free area as in Figure 2D was obtained by manually drawing the outline of the ribosome free area in each partner cell at the zone of cell-cell contact on one tomogram virtual slice and measuring its surface.

FRAP data analysis was performed by recording the fluorescence intensity of the bleached area using a manually fitted ROI, which was occasionally moved to track moving foci, which we could follow through the whole time-lapse as we only partially bleached the observed structures. Cells where the Fus1 foci could not be followed over the entire time course of the time lapse were excluded from the analysis. All the remaining traces were background substracted and bleach-corrected as above.

For Figure 1I, they were then scaled from minimum to pre-bleaching value as follows:

$$Traces_{Scaled} = \frac{Traces_t - Traces_{t=1\ post\ bleaching}}{Mean\left(Traces_{pre\ bleaching}\right) - Traces_{t=1\ post\ bleaching}}$$

For Figure 4G, they were scaled from minimum to maximum recovery value as follows:

$$Traces_{Scaled} = \frac{Traces - Traces_{t=1\ post\ bleaching}}{Max(Traces) - Traces_{t=1\ post\ bleaching}}$$

The resulting scaled traces were then averaged for each condition. These average traces were then used to fit the following conventional FRAP equation for each replicate and each condition:

$$f(t) = A\left(1 - e^{-\tau t}\right)$$

In the three replicates performed for Figure 1I, we obtained the following R^2: 0.9844, 0.9917 and 0.9881 for Fus1N-Tips, 0.9534, 0.9683 and 0.96690 for Fus1N-Clusters. For the replicates performed for Figure 4G, we obtained the following R^2: 0.8836, 0.9349, 0.9674 and 0.9538 for WT, 0.8698, 0.9801 and 0.9715 for $fus1^{\Delta IDR}$, 0.9466 and 0.9349 for FUS^{12E}, 0.9364 and 0.9869 for FUS, 0.9141 and 0.9677 for $CRY2^{PHR}$ and 0.9135 and 0.9595 for $CRY2^{olig}$. We used the fitted value of τ to calculate the half-time of recovery $\tau_{1/2}$ as follow:

$$\tau_{1/2} = \frac{\ln(0.5)}{\tau}$$

As we made several replicates, we then obtained several values per condition, which were averaged and indicated directly on the figure along with their standard deviation, or were plotted independently into a boxplot (Figure 3H). The graphs show the average from the first post-beaching point of all traces from all replicates for each condition along with their standard error.

Fiducial-based correlation was done using the Icy plug-in eC-CLEM,[53] through 2D rigid transformation and manual matching of features. First correlation between light microscopy and low magnification electron microscopy images or tomograms (with different pixel sizes depending on the field of view that was required to have a sufficient number of fiducials) was done using TetraSpeck beads, which are visible in both images. The resulting overlay images were then correlated with high magnification tomograms using 15-nm protein A–coupled gold beads as fiducials.

All plots, fittings, corrections and normalisations were made using MATLAB home-made scripts. For boxplots, the central line indicates the median, and the bottom and top edges of the box indicate the 25th and 75th percentiles, respectively. The whiskers extend to the most extreme data points not considered outliers. For bar plots, error bars represent the standard deviation. For the two FRAP plots, shaded areas represent the standard error. Statistical p-values were obtained using a two-sided student's t-test, after normal distribution had been visually checked using a simple histogram. No further verification was made to ascertain that the data met assumptions of the statistical approach. All values below 0.05 are mentioned in the figures, including sample size. In all figures, N indicates the number of independent experiments, and n the number of events quantified in each experiment.

Current Biology, Volume *32*

Supplemental Information

Condensation of the fusion focus by the intrinsically disordered region of the formin Fus1 is essential for cell-cell fusion

Ingrid Billault-Chaumartin, Olivia Muriel, Laetitia Michon, and Sophie G. Martin

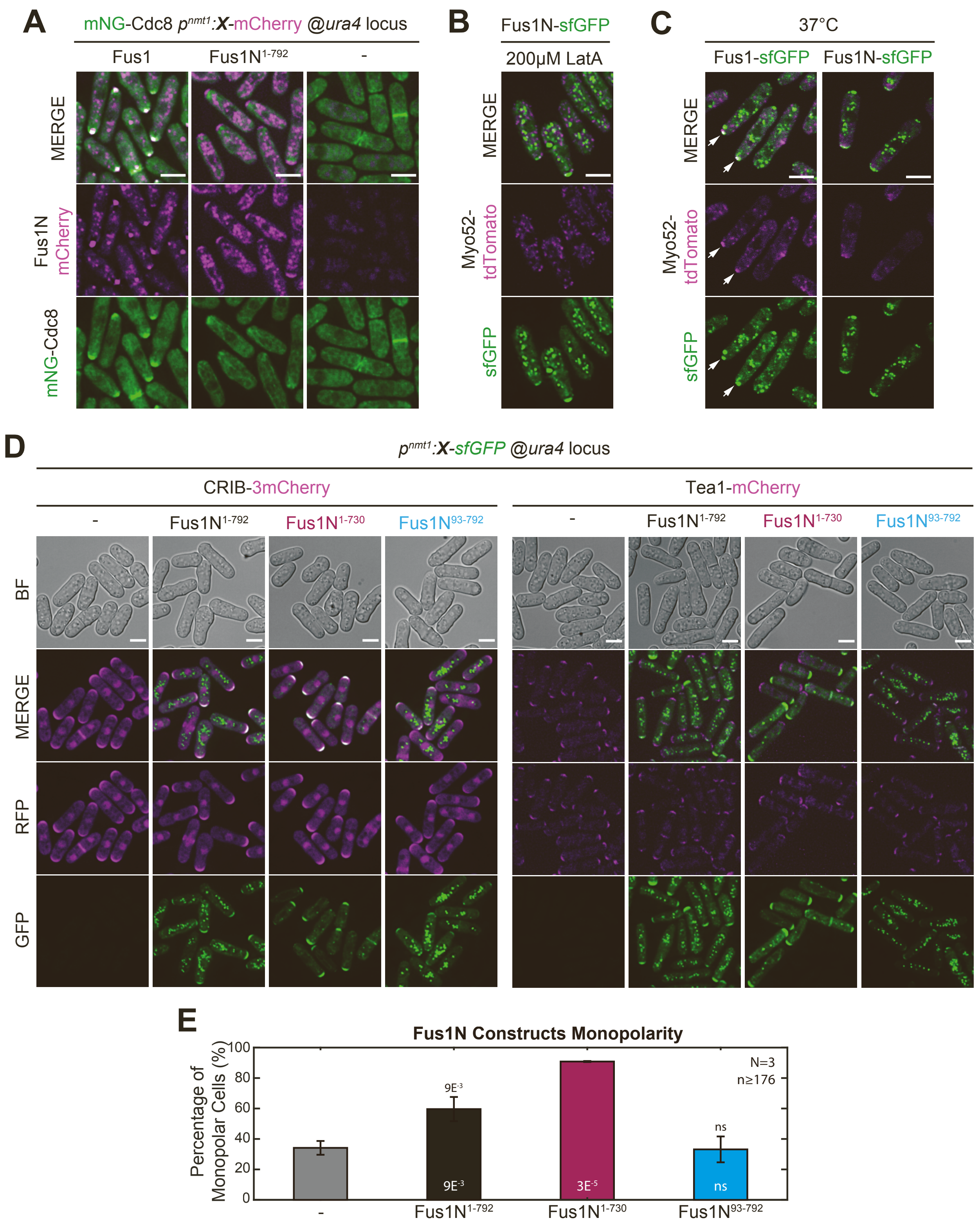

Figure S1 . Additional localization data and Fus1N tip localization effect on polarity. Related to Figure 1.

A. Interphase cells expressing mNeonGreen-Cdc8, either in combination with (left) full length Fus1-sfGFP or (middle) Fus1N-sfGFP (Fus1$^{1-792}$) or (right) alone. **B.** Interphase cells expressing Myo52-tdTomato and Fus1N-sfGFP (Fus1$^{1-792}$). Cells were treated with 200μM LatrunculinA for 5 minutes. **C.** Interphase cells expressing Myo52-tdTomato and either (left) full length Fus1-sfGFP or (right) Fus1N-sfGFP (Fus1$^{1-792}$) grown for 6h at 37°C and imaged at 40°C. White arrows mark resistant fusion focus-like structure. **D.** DIC

and fluorescence images of either (left) the polarity marker CRIB-3mCherry or (right) Tea1-mCherry in interphase WT or Fus1N$^{1-792}$, Fus1N$^{1-730}$ or Fus1N$^{93-792}$-expressing cells. **E.** Monopolarity of the strains as in (D), assessed from the localization of CRIB on a single snapshot. Of note, a fraction of WT cells appear monopolar using this assay, because they are either before NETO or at a time point in CRIB oscillations [52] where only one tip is decorated. All p-values are relative to WT. Bars are 5µm.

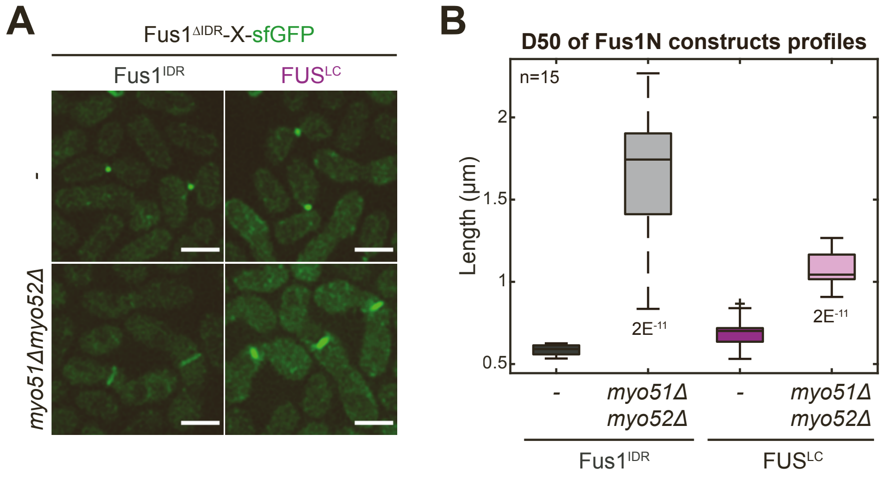

Figure S2. Type V myosins are required for fusion focus focalization in both WT cells and cells in which Fus1 IDR was replaced by FUS^{LC}. Related to Figure 4.

A. Images of (left) Fus1-sfGFP or (right) Fus1$^{1-491}$-FUS^{LC}-Fus1$^{792-1372}$-sfGFP in mating cell pairs that are otherwise WT (top) or carry deletions of *myo51Δ* and *myo52Δ* (bottom). **B.** Width at half maximum of GFP-fluorescence profiles in strains as in (A). p-values compare WT Fus1 and Fus1$^{1-491}$-FUS^{LC}-Fus1$^{792-1372}$ in each background. Bars are 5µm.

Table S1: Strains used in this study and links to figures. Related to STAR Methods.

GENOTYPE	FIGURES	STRAIN
h90 myo52-tdTomato:natMX fus1-sfGFP:kanMX ura4- leu1-32 ade6-M216	1, 4, S2	YSM3312
h90 myo52-tdTomato:natMX fus1Δ::LEU2+ ura4-294:p^{fus1}-fus1N^{1-792}-fus1$C^{793-1372}$-sfGFP:ura4+ leu1-32	1	YSM2504
h90 myo52-tdTomato:natMX fus1Δ::LEU2+ ura4-294:p^{fus1}-cdc12N^{1-887}-fus1$C^{793-1372}$-sfGFP:ura4+ leu1-32	1	YSM2512
h90 myo52-tdTomato:natMX fus1Δ::LEU2+ ura4-294:p^{fus1}-for3N^{1-714}-fus1$C^{793-1372}$-sfGFP:ura4+ leu1-32	1	YSM2510
h90 myo52-tdTomato:natMX ura4+:p^{nmt1}:fus1N^{1-792}-sfGFP:$term^{nmt}$ leu1-32 ade6-M210	1, 3, S1	YSM4002
h90 myo52-tdTomato:natMX ura4+:p^{nmt1}:fus1N^{1-730}-sfGFP:$term^{nmt}$ leu1-32 ade6-M210	1, 3	YSM4003
h90 myo52-tdTomato:natMX ura4+:p^{nmt1}:fus1N^{1-500}-sfGFP:$term^{nmt}$ leu1-32 ade6-M210	1, 3	YSM4004
h90 myo52-tdTomato:natMX ura4+:p^{nmt1}:fus1N^{93-792}-sfGFP:$term^{nmt}$ leu1-32 ade6-M210	1	YSM4005
h90 myo52-tdTomato:natMX ura4+:p^{nmt1}:fus1$N^{140-792}$-sfGFP:$term^{nmt}$ leu1-32 ade6-M210	1	YSM4006
h90 myo52-tdTomato:natMX ura4+:p^{nmt1}:fus1$N^{191-792}$-sfGFP:$term^{nmt}$ leu1-32 ade6-M210	1, 3	YSM4007
h90 myo52-tdTomato:natMX ura4+:p^{nmt1}:fus1$N^{431-755}$-sfGFP:$term^{nmt}$ leu1-32 ade6-M210	1	YSM4008
h90 myo52-tdTomato:natMX ura4+:p^{nmt1}:fus1-sfGFP:$term^{nmt}$ leu1-32 ade6-M210	1, S1	YSM4009
h90 leu1-32:p^{cdc8}:mNeonGreen-cdc8:$term^{cdc8}$:$term^{ScADH1}$:leu1+ ura4-D18 ade6-M216	S1	YSM3786
h90 ura4+:p^{nmt1}:fus1-mCherry:$term^{nmt}$ leu1-32:p^{cdc8}:mNeonGreen-cdc8:$term^{cdc8}$:$term^{ScADH1}$:leu1+ ade6-M216	S1	YSM4042
h90 ura4+:p^{nmt1}:fus1N^{1-792}-mCherry:$term^{nmt}$ leu1-32:pcdc8:mNeonGreen-cdc8:$term^{cdc8}$:$term^{ScADH1}$:leu1+ ade6-M216	S1	YSM4043
h+ his5+:p^{act1}:CRIB-3mCherry:bsdMX ura4-D18	S1	YSM4010
h90 his5+:p^{act1}:CRIB-3mCherry:bsdMX ura4+:p^{nmt1}:fus1N^{1-792}-sfGFP:$term^{nmt}$ leu1-32	S1	YSM4011
h90 his5+:p^{act1}:CRIB-3mCherry:bsdMX ura4+:p^{nmt1}:fus1N^{1-730}-sfGFP:$term^{nmt}$ ade6-M210	S1	YSM4012
h90 his5+:p^{act1}:CRIB-3mCherry:bsdMX ura4+:p^{nmt1}:fus1N^{93-792}-sfGFP:$term^{nmt}$ leu1-32 ade6-M210	S1	YSM4013
h90 tea1-mCherry:kanMX ura4-D18 leu1-32	S1	YSM4014
h90 tea1-mCherry:kanMX ura4+:p^{nmt1}:fus1N^{1-792}-sfGFP:$term^{nmt}$ leu1-32	S1	YSM4015
h90 tea1-mCherry:kanMX ura4+:p^{nmt1}:fus1N^{1-730}-sfGFP:$term^{nmt}$ leu1-32	S1	YSM4016
h90 tea1-mCherry:kanMX ura4+:p^{nmt1}:fus1N^{93-792}-sfGFP:$term^{nmt}$ leu1-32 ade6-M210	S1	YSM4017
h90 myo52-tdTomato:natMX fus1-sfGFP:kanMX	2	YSM3888
h90 myo52-tdTomato:natMX fus1-sfGFP:kanMX acp2Δ::bleMX ura4- leu1-32 ade6-M210	2	YSM3314
h90 myo52-tdTomato:natMX ura4+:p^{nmt1}:fus1N^{93-792}-sfGFP:$term^{nmt}$ fus1Δ::hphMX ade6-M210 leu1-32	2	YSM4018
h90 myo52-tdTomato:natMX ura4+:p^{nmt1}:fus1-sfGFP:$term^{nmt}$ fus1Δ::hphMX ade6-M210 leu1-32	2	YSM4053
h90 myo52-tdTomato:natMX ura4-294:p^{fus1}:fus1N-sfGFP:ura4+ fus1Δ::LEU2+ leu1-32	3	YSM2486
h90 myo52-tdTomato:natMX ura4-294:p^{fus1}:fus1N-sfGFP:ura4+ leu1-32	3	YSM2699
h90 myo52-tdTomato:natMX ura4+:p^{nmt1}:fus1N^{1-792}-sfGFP:$term^{nmt}$ fus1Δ::hphMX leu1-32 ade6-M210	3	YSM4054
h90 myo52-tdTomato:natMX ura4+:p^{nmt1}:fus1$N^{191-792}$-sfGFP:$term^{nmt}$ fus1Δ::hphMX leu1-32 ade6-M210	3	YSM4055
h90 myo52-tdTomato:natMX ura4+:p^{nmt1}:fus1N^{1-730}-sfGFP:$term^{nmt}$ fus1Δ::hphMX leu1-32 ade6-M210	3	YSM4056
h90 myo52-tdTomato:natMX ura4+:p^{nmt1}:fus1N^{1-500}-sfGFP:$term^{nmt}$ fus1Δ::hphMX leu1-32 ade6-M210	3	YSM4057
h90 myo52-tdTomato:natMX $fus1^{\Delta 501-749}$-sfGFP:kanMX ura4-294 leu1-32 ade6-M210	3	YSM4019
h90 myo52-tdTomato:natMX $fus1^{\Delta 501-791}$-sfGFP:kanMX ura4-294 leu1-32 ade6-M210	3	YSM4020
h90 myo52-tdTomato:natMX $fus1^{\Delta 492-791}$-sfGFP:kanMX ura4-294 leu1-32 ade6-M210	3, 4	YSM4021
h90 myo52-tdTomato:natMX $fus1^{\Delta 492-500}$-sfGFP:kanMX ura4-294 leu1-32 ade6-M210	3	YSM4044
h90 myo52-tdTomato:natMX $fus1^{\Delta 731-791}$-sfGFP:kanMX ura4-294 leu1-32 ade6-M210	3	YSM4045
h90 myo52-tdTomato:natMX $fus1^{\Delta 492-749}$-sfGFP:kanMX ura4-294 leu1-32 ade6-M210	3	YSM4046
h90 myo52-tdTomato:natMX $fus1^{1-491}$-FUS^{12E}-$fus1^{792-1372}$-sfGFP:kanMX ura4-294 leu1-32 ade6-M210	4	YSM4022
h90 myo52-tdTomato:natMX $fus1^{1-491}$-FUS-$fus1^{792-1372}$-sfGFP:kanMX ura4-294 leu1-32 ade6-M210	4, S2	YSM4023
h90 myo52-tdTomato:natMX $fus1^{1-491}$-$CRY2^{PHR}$-$fus1^{792-1372}$-sfGFP:kanMX ura4-294 leu1-32 ade6-M210	4	YSM4024
h90 myo52-tdTomato:natMX $fus1^{1-491}$-$CRY2^{olig}$-$fus1^{792-1372}$-sfGFP:kanMX ura4-294 leu1-32 ade6-M210	4	YSM4025
h90 myo52-tdTomato:natMX $fus1^{1-491}$-FUS^{G156E}-$fus1^{792-1372}$-sfGFP:kanMX ura4-294 leu1-32 ade6-M210	4	YSM4047
h90 myo52-tdTomato:natMX leu1-32:p^{cdc8}:mNeonGreen-cdc8:$term^{cdc8}$:$term^{ScADH1}$:leu1+ fus1:kanMX ura4-294 ade6-M210	4	YSM4026
h90 myo52-tdTomato:natMX leu1-32:p^{cdc8}:mNeonGreen-cdc8:$term^{cdc8}$:$term^{ScADH1}$:leu1+ $fus1^{1-491}$-$CRY2^{PHR}$-$fus1^{792-1372}$:kanMX ura4-294 ade6-M210	4	YSM4048
h90 myo52-tdTomato:natMX leu1-32:p^{cdc8}:mNeonGreen-cdc8:$term^{cdc8}$:$term^{ScADH1}$:leu1+ $fus1^{1-491}$-$CRY2^{olig}$-$fus1^{792-1372}$:kanMX ura4-294 ade6-M210	4	YSM4049
h90 myo52-tdTomato:natMX leu1-32:p^{cdc8}:mNeonGreen-cdc8:$term^{cdc8}$:$term^{ScADH1}$:leu1+ $fus1^{1-491}$-FUS-$fus1^{792-1372}$:kanMX ura4-294 ade6-M210	4	YSM4050
h90 myo52-tdTomato:natMX leu1-32:p^{cdc8}:mNeonGreen-cdc8:$term^{cdc8}$:$term^{ScADH1}$:leu1+ $fus1^{1-491}$-FUS^{12E}-$fus1^{792-1372}$:kanMX ura4-294 ade6-M210	4	YSM4051
h90 fus1-sfGFP:kanMX myo51Δ::ura4+ myo52Δ::ura4+ leu1-32	S2	YSM2543
h90 $fus1^{1-491}$-FUS-$fus1^{792-1372}$-sfGFP:kanMX myo51Δ::ura4+ myo52Δ::ura4+ leu1-32	S2	YSM4052

Table S2: Primers used in this study and their usage

HR stands for homologous recombination in yeast [45] and SDM for site directed mutagenesis

NAME	SEQUENCE	ORIENTATION	PURPOSE
osm765	CAGCTCCAAATTTTGAAAGTAAAACCCCTAATTAGGGAATAAATAAGTAGGCAGAGCAC CTTGAAAAATAACTAGATAGAATTCGAGCTCGTTTAAAC	R	HR (Myo52 3’)
osm932	AATAAAAAGAGACAAACAGTCGTCCTTAAAGCTGAATGCATGCTTAAGCAGCTGGAGA ATAACAATGAACTTAAGAGACGGATCCCCGGGTTAATTAA	F	HR (Fus1 ORF)
osm933	TTTTATTAATTATAATTTCATTATAATTTGTTTAAGTCATTTAATTGTCATTAAAAGTCATT AACATTTCAAACATCAGAATTCGAGCTCGTTTAAAC	R	HR (Fus1 3’)
osm1196	GATCACTGTAGGCAACGTAGCCGACAATGATGTACAGAACTCGAGCGACGAAGAAAAT CAAGTACCAAATGGTATTAAAGTTCGGATCCCCGGGTTAATTAA	F	HR (Myo52 ORF)
osm1746	ACGGATTTCATGAAGTTATTGGTTAAAAGCGGCCTCTCAAATCCTCCAGCTAAAGAACCA GTCCATGACAACGAAAATCGGATCCCCGGGTTAATTAA	F	HR (Tea1 ORF)
osm1747	ATGTCATCGTCGAATATTTACACTATGTACAGTCCTTTCAACTAGTAAAGGAGATGCTTT CAAAATAGTTCCAAAGAGGAATTCGAGCTCGTTTAAAC	R	HR (Tea1 3’)
osm1772	CGTATCACGAGGCCCTTTCG	F	CLONING
osm2217	CCGGATCCTCCAAGGGTGAAGAGCTATTTACTGGGG	F	CLONING
osm3005	ACTGCGGCCGCATGATGACGGCTAGTTTTAAAGG	F	CLONING
osm3006	ACTCCCGGGTCTCTTAAGTTCATTGTTATTCTCC	R	CLONING
osm3007	ACTGCGGCCGCATGGCATCTAAAATGCCTGAAG	F	CLONING
osm3009	ACTGCGGCCGCATGCGAAATTCGTCAAAGGGAC	F	CLONING
osm3026	CTTGGATCCTCATATTTTCTATTTTAGAAAACCTC	R	CLONING
osm3027	TGAGGATCCAAGAAGTTATTGATGGGAATCC	F	CLONING
osm3028	CTGGGATCCATGGCGAAGGCGAGGAAG	R	CLONING
osm3030	TCGGGATCCTACTATTGTTGCTAACTGTTTCTGC	R	CLONING
osm3031	GTAGGATCCCGAACTTTGATATTCCTAATGATGC	F	CLONING
osm3091	CGGGGTACCGATCAGAAAATTATCGCCAT	F	CLONING
osm3516	ACTGCGGCCGCTGATTTAACAAAGCGACTATAAGTC	R	CLONING
osm3521	ACTCCCGGGAGTAGAAGTGTTAGGAGCTTC	R	CLONING
osm4021	CTTGGATCCTATGAACCTCAAAAGAATGCGTTG	R	CLONING
osm4504	CATTAAGGCCTCACTTTTATTCTGAGATCGCTATCCGGTTGTATTCTTTTGTTTAAAGCAT TATATCATCAACTCACCCGGATCCCCGGGTTAATTAA	F	HR (Acp2 5’)
osm4505	CAATCTTTCTATGACTATTTTCGTTGAAGATGGAACGAATACTATGAGAAGATCACGGAA AGAAAACAAAAAGCAATCGAATTCGAGCTCGTTTAAAC	R	HR (Acp2 3’)
osm4577	GGAATAAGGGCGACACGG	F	ANALYTICAL
osm5452	GGCCACTAGTGGATCTGATATCGATGTATTTACTGATTACTT	R	INFUSION
osm5453	CTTCTAAACGGCTAGCTCAGCTTCATTGG	F	SDM
osm5454	CAATGAAGCTGAGCTAGCCGTTTAGAAGG	R	SDM
osm6064	CATATGGTCTGGGTATCT	R	CLONING
osm6183	GCCTTCCAACCAGCTTCTCT	R	ANALYTICAL
osm6576	CTTGGATCCATCATTATTTGAATTACCAT	R	CLONING
osm6582	CTTGTTTAAACCAACATGCCTGTAAG	R	CLONING
osm6583	GAAGTTTAAACTGCTTTTGTGGTTATC	F	CLONING
osm7119	CTTCGTACGCTGCAGGTCGACACAGTATGTACGCCAC	F	INFUSION
osm7122	TTCACCCTTGGAGTTAATTAATCTCTTAAGTTCATTGTTAT	R	INFUSION
osm7127	ATGTACCAGGCGAAGCGCTTCTATGTCCGGATGAC	F	INFUSION
osm7140	CTTCTTTGATTCTCATATCAGCTTGTAAAGTAAGC	R	INFUSION
osm7141	TACTTTACAAGCTGATATGAGAATCAAAGAAGTTAT	F	INFUSION
osm7204	CTTTGTTAAATCAGCGGCCGCATGTTTACCGATTCATATGTA	F	INFUSION
osm7205	CTTGGAGTTAATTAACCCGGGGATCCTCATATTTTC	R	INFUSION
osm7254	GCTTTGTTAAATCAGCGGCCGCATGATGAC	F	INFUSION
osm7255	CTTGGAGTTAATTAACCCGGGGATCCTATCATTATTTGAATTACCA	R	INFUSION
osm7256	CTTTGTTAAATCAGCGGCCGCATGAAGCACACTCCAAATTCT	F	INFUSION
osm7257	CTTGGAGTTAATTAACCCGGGGATCCTAAAAACCTTGTGTTTTGA	R	INFUSION
osm7487	CTTCTTTGATTCTCATATCATTATTTGAATTACCAT	R	INFUSION
osm7488	TAATTCAAATAATGATATGAGAATCAAAGAAGTTAT	F	INFUSION
osm7489	AAACCTTGTGTTTTGAATCAGCTTGTAAAGTAAG	R	INFUSION
osm7490	TACTTTACAAGCTGATTCAAAACACAAGGTTTTTA	F	INFUSION
osm7499	GCTTTGTTAAATCAGCGGCCGCATGCTCAAGTACGTGGAATCTTT	F	INFUSION

osm7638	CTTTGTTAAATCAGCGGCCGCATGGTTACACTCTCTCAAGAAAA	F	INFUSION
osm7677	GGAGTATTAAAACAACTCGAGAAATGCGTGAAACTC	F	INFUSION
osm7690	AAATCAAGGATATGAGAATTCCGAAAGAAAGTATGT	F	INFUSION
osm7738	TATAAAAGCAATCAATATCAGCTTGTAAAGTAAGCAC	R	INFUSION
osm7739	TACTTTACAAGCTGATATTGATTGCTTTTATAAGGAATTAAAG	F	INFUSION
osm7740	GCTTATTTAGAAGTGGCGCGCCTCTCTTAAGTTCATTGTTATTC	R	INFUSION
osm7875	CTTCTTTGATTCTCATATGAACCTCAAAAGAATGCG	R	INFUSION
osm7876	TTCTTTTGAGGTTCATATGAGAATCAAAGAAGTTATTGAT	F	INFUSION
osm7877	CTTCTGATTTACAGTGCTAGCCTTTTTGTACTCCAGTATTAT	R	INFUSION
osm7878	TTTTGTCCATCTTCATCGTCATCATTAACAAGCAATAG	R	INFUSION
osm7879	CTTGTTAATGATGACGATGAAGATGGACAAAAAGACTAT	F	INFUSION
osm7880	AACTAGCCGTCATCATTGCTGCTCCGATCATGATCT	R	INFUSION
osm7881	CATGATCGGAGCAGCAATGATGACGGCTAGTTTTAAAG	F	INFUSION
osm7882	GAGTTTCACGCATTTCTCGAGTTGTTTTAATACTCCTTC	R	INFUSION
osm8388	ACTGGTTCTGCTGTTCATAGCCCTGAGGGGGATTA	R	INFUSION
osm8389	CCCTCAGGGCTATGAACAGCAGAACCAGTACAAC	F	INFUSION
osm8480	ACTTAAGAGAGGATCCCCGGGTTAATTAAC	F	INFUSION
osm8481	ATTCCTTTTACCCGGTTTACTTGTACAGCTCGTCC	R	INFUSION
osm8482	CGAGCTGTACAAGTAAACCGGGTAAAAGGAATGTC	F	INFUSION
osm8483	AGGGAACAAAAGCTGGAGC	R	INFUSION
osm8484	GAAAATATGAGGATCCCCGGGTTAATTAAC	F	INFUSION

Table S3: Plasmids used in this study and their construction. Related to STAR Methods.

For each plasmid, the column “obtained from” indicates how it was constructed, from restriction enzyme-based cloning or infusion, with the primers and restriction enzymes used. “WT” indicates that genomic DNA from a wildtype strain was used as template for PCR amplification.

NAME	DESCRIPTION	OBTAINED FROM	USAGE
pAV133	pUra4^{AfeI}	[46]	Single integration at *ura4*
pSM617	pREP3x	Lab Stock	Pombe expression
pSM677	pFA6a-mCherry-kanMX	Lab Stock	template for PCR-based HR
pSM684	pFA6a-mCherry-natMX	Lab Stock	template for PCR-based HR
pSM685	pFA6a-tdTomato-natMX	Lab Stock	template for PCR-based HR
pSM694	pFA6a-bleMX	Lab Stock	template for PCR-based HR
pSM1538	pFA6a-sfGFP-kanMX	Lab Stock	template for PCR-based HR
pSM1638	pRIP-p^{fus1}-sfGFP	Lab Stock	Multiple integration at *ura4*
pSM1650	pRIP-p^{fus1}-fus1N-sfGFP	Regular cloning :<br>pSM1638$^{NotI/BamHI}$+(WT$^{osm3005-osm3026}$)$^{NotI/BamHI}$	Multiple integration at *ura4*
pSM1656	pRIP-p^{fus1}-fus1-sfGFP	Regular cloning :<br>pSM1638$^{NotI/XmaI}$+(WT$^{osm3005-osm3006}$)$^{NotI/XmaI}$	Multiple integration at *ura4*
pSM1659	pRIP-p^{fus1}-fus1N-fus1C-sfGFP	3-point ligation cloning :<br>pSM1638$^{NotI/XmaI}$+(WT$^{osm3005-osm3026}$)$^{NotI/BamHI}$<br>+(WT$^{osm3027-osm3006}$)$^{BamHI/XmaI}$	Multiple integration at *ura4*
pSM1662	pRIP-p^{fus1}-for3N-fus1C-sfGFP	3-point ligation cloning :<br>pSM1638$^{NotI/XmaI}$+(WT$^{osm3007-osm3028}$)$^{NotI/BamHI}$<br>+(WT$^{osm3027-osm3006}$)$^{BamHI/XmaI}$	Multiple integration at *ura4*
pSM1663	pRIP-p^{fus1}-cdc12N-fus1C-sfGFP	3-point ligation cloning :<br>pSM1638$^{NotI/XmaI}$+(WT$^{osm3009-osm3030}$)$^{NotI/BamHI}$<br>+(WT$^{osm3027-osm3006}$)$^{BamHI/XmaI}$	Multiple integration at *ura4*
pSM1823	pRIP-p^{nmt41}-sfGFP	Lab Stock	Multiple integration at *ura4*
pSM1826	pRIP-p^{nmt41}-fus1N-sfGFP	Subcloning :<br>pSM1650$^{KpnI/NotI}$+pSM1823$^{KpnI/NotI}$	Multiple integration at *ura4*
pSM2229	pUra4^{AfeI}-p^{nmt41}-fus1-sfGFP	Lab Stock, Derived from pAV133	Single integration at *ura4*
pSM2251	pFA6a-fus1$^{5'UTR}$-fus1_K879A-sfGFP-kanMX-fus1$^{3'UTR}$	SDM :<br>pSM2827$^{osm5453/5454}$	Single integration at endogenous *fus1* locus
pSM2390	pRIP-p^{fus1}-CRY2olig-For3N-fus1C-sfGFP	Lab Stock, Derived from pAV133	Multiple integration at *ura4*
pSM2475	pUra4^{AfeI}-p^{fus1}-CRY2PHR-fus1C-sfGFP	Lab Stock, Derived from pSM1662	Single integration at *ura4*
pSM2478	pUra4^{PmeI}-p^{nmt41}-fus1-sfGFP	3-point ligation cloning :<br>pSM2229$^{AatII/StuI}$+(pSM2229$^{osm4577-osm6582}$)$^{AatII/PmeI}$<br>+(pSM2229$^{osm6583-osm6183}$)$^{PmeI/StuI}$	Single integration at *ura4*
pSM2507	pFA6a-fus1$^{5'UTR}$-fus1$^{\Delta 501-749}$-sfGFP-kanMX-fus1$^{3'UTR}$	3-point ligation cloning :<br>pSM2251$^{SalI/PacI}$+(pSM2251$^{osm1772-osm6576}$)$^{SalI/BamHI}$<br>+(pSM2229$^{osm3031-osm3521}$)$^{BamHI/PacI}$	Single integration at endogenous *fus1* locus
pSM2600	pUra4^{PmeI}-p^{nmt1}-fus1N-sfGFP	3-point ligation cloning :<br>pSM2478$^{KpnI/SacI}$+(pSM617$^{osm3091-osm3516}$)$^{KpnI/NotI}$<br>+pSM1826$^{NotI/SacI}$	Single integration at ura4
pSM2601	pUra4^{PmeI}-p^{nmt1}-fus1N$^{1-730}$-sfGFP	3-point ligation cloning:<br>pSM2600$^{NotI/MscI}$+(pSM2600$^{osm3005-osm4021}$)$^{NotI/BamHI}$+(pSM2600$^{osm2217-osm6064}$)$^{BamHI/MscI}$	Single integration at *ura4*
pSM2602	pUra4^{PmeI}-p^{nmt1}-fus1-sfGFP	Subcloning :<br>pSM2600$^{NotI/XmaI}$+pSM1656$^{NotI/XmaI}$	Single integration at *ura4*
pSM2625	pFA6a-fus1$^{5'UTR}$-fus1$^{\Delta 492-791}$-sfGFP-kanMX-fus1$^{3'UTR}$	Infusion cloning :<br>pSM2507$^{SalI/PacI}$+WT$^{osm7119-osm7140}$ +WT$^{osm7141-osm7122}$	Single integration at endogenous *fus1* locus
pSM2630	pUra4^{PmeI}-p^{nmt1}-fus1N$^{93-792}$-sfGFP	Infusion cloning :<br>pSM2600$^{NotI/XmaI}$+pSM2600$^{osm7204-osm7205}$	Single integration at *ura4*
pSM2644	pUra4^{PmeI}-p^{nmt1}-fus1N$^{1-500}$-sfGFP	Infusion cloning :<br>pSM2600$^{NotI/XmaI}$+pSM2600$^{osm7254-osm7255}$	Single integration at *ura4*
pSM2645	pUra4^{PmeI}-p^{nmt1}-fus1N$^{431-755}$-sfGFP	Infusion cloning :<br>pSM2600$^{NotI/XmaI}$+pSM2600$^{osm7256-osm7257}$	Single integration at *ura4*

pSM2697	pFA6a-fus1$^{5'UTR}$-fus1$^{\Delta 501\text{-}791}$-sfGFP-kanMX-fus1$^{3'UTR}$	Infusion cloning : pSM2507$^{SalI/PacI}$+WT$^{osm7119\text{-}osm7487}$ +WT$^{osm7488\text{-}osm7122}$	Single integration at endogenous *fus1* locus
pSM2698	pFA6a-fus1$^{5'UTR}$-fus1$^{\Delta 492\text{-}749}$-sfGFP-kanMX-fus1$^{3'UTR}$	Infusion cloning : pSM2507$^{SalI/PacI}$+WT$^{osm7119\text{-}osm7489}$ +WT$^{osm7490\text{-}osm7122}$	Single integration at endogenous *fus1* locus
pSM2703	pUra4^{PmeI}-p^{nmt1}-fus1N$^{191\text{-}792}$-sfGFP	Infusion cloning : pSM2600$^{NotI/XmaI}$+pSM2600$^{osm7499\text{-}osm7205}$	Single integration at *ura4*
pSM2825	pUra4^{PmeI}-p^{nmt1}-fus1N$^{140\text{-}792}$-sfGFP	Infusion cloning : pSM2600$^{NotI/XmaI}$+pSM2600$^{osm7638\text{-}osm7205}$	Single integration at *ura4*
pSM2827	pFA6a-fus1$^{5'UTR}$-fus1-sfGFP-kanMX-fus1$^{3'UTR}$	Infusion cloning : pSM1538$^{SalI/EcoRV}$+IBC180$^{osm7119\text{-}osm5452}$	Single integration at endogenous *fus1* locus
pSM2912	pFA6a-fus1$^{5'UTR}$-fus1$^{\Delta 492\text{-}500}$-sfGFP-kanMX-fus1$^{3'UTR}$	Infusion cloning : pSM2507$^{AfeI/PacI}$+pSM2507$^{osm7127\text{-}osm7738}$ +pSM2507$^{osm7739\text{-}osm7122}$	Single integration at endogenous *fus1* locus
pSM2913	pFA6a-fus1$^{5'UTR}$-fus1-kanMX-fus1$^{3'UTR}$	Infusion cloning : pSM2827$^{EcoRI/AscI}$+pSM2827$^{osm7690\text{-}osm7740}$	Single integration at endogenous *fus1* locus
pSM2937	pFA6a-fus1$^{5'UTR}$-fus1$^{1\text{-}491}$-CRY2PHR-fus1$^{792\text{-}1372}$-sfGFP-kanMX-fus1$^{3'UTR}$	Infusion cloning : pSM2625$^{SalI/XhoI}$+pSM2625$^{osm7119\text{-}osm7878}$ +pSM2475$^{osm7879\text{-}osm7880}$+pSM2625$^{osm7881\text{-}osm7882}$	Single integration at endogenous *fus1* locus
pSM2938	pFA6a-fus1$^{5'UTR}$-fus1$^{1\text{-}491}$-CRY2olig-fus1$^{792\text{-}1372}$-sfGFP-kanMX-fus1$^{3'UTR}$	Infusion cloning : pSM2625$^{SalI/XhoI}$+pSM2625$^{osm7119\text{-}osm7878}$ +pSM2390$^{osm7879\text{-}osm7880}$+pSM2625$^{osm7881\text{-}osm7882}$	Single integration at endogenous *fus1* locus
pSM2939	pFA6a-fus1$^{5'UTR}$-fus1$^{\Delta 731\text{-}791}$-sfGFP-kanMX-fus1$^{3'UTR}$	Infusion cloning : pSM2827$^{XhoI/NheI}$+pSM2827$^{osm7677\text{-}osm7875}$ +pSM2827$^{osm7876\text{-}osm7877}$	Single integration at endogenous *fus1* locus
pSM2940	pFA6a-fus1$^{5'UTR}$-fus1$^{1\text{-}491}$-FUS-fus1$^{792\text{-}1372}$-sfGFP-kanMX-fus1$^{3'UTR}$	Infusion cloning : pSM2625$^{XhoI/SwaI}$+fus1$^{XhoIsite\text{-}491}$-FUS-fus1$^{792\text{-}SwaIsite}$ ordered as a gBlock	Single integration at endogenous *fus1* locus
pSM2941	pFA6a-fus1$^{5'UTR}$-fus1$^{1\text{-}491}$-FUS^{12E}-fus1$^{792\text{-}1372}$-sfGFP-kanMX-fus1$^{3'UTR}$	Infusion cloning : pSM2625$^{XhoI/SwaI}$+fus1$^{XhoIsite\text{-}491}$-FUS^{12E}-fus1$^{792\text{-}SwaIsite}$ ordered as a gBlock	Single integration at endogenous *fus1* locus
pSM3032	pFA6a-fus1$^{5'UTR}$-fus1$^{1\text{-}491}$-FUS^{G156E}-fus1$^{792\text{-}1372}$-sfGFP-kanMX-fus1$^{3'UTR}$	Infusion cloning : pSM2827$^{XhoI/NheI}$+pSM2940$^{osm7677\text{-}osm8388}$ +pSM2940$^{osm8389\text{-}osm7877}$	Single integration at endogenous *fus1* locus
pSM3034	pFA6a-fus1$^{5'UTR}$-fus1$^{1\text{-}491}$-CRY2PHR-fus1$^{792\text{-}1372}$-kanMX-fus1$^{3'UTR}$	Subcloning : pSM2913$^{SalI/NheI}$+pSM2937$^{NotI/XmaI}$	Single integration at endogenous *fus1* locus
pSM3035	pFA6a-fus1$^{5'UTR}$-fus1$^{1\text{-}491}$-CRY2olig-fus1$^{792\text{-}1372}$-kanMX-fus1$^{3'UTR}$	Subcloning : pSM2913$^{SalI/NheI}$+pSM2938$^{NotI/XmaI}$	Single integration at endogenous *fus1* locus
pSM3036	pFA6a-fus1$^{5'UTR}$-fus1$^{1\text{-}491}$-FUS-fus1$^{792\text{-}1372}$-kanMX-fus1$^{3'UTR}$	Subcloning : pSM2913$^{SalI/NheI}$+pSM2940$^{NotI/XmaI}$	Single integration at endogenous *fus1* locus
pSM3037	pFA6a-fus1$^{5'UTR}$-fus1$^{1\text{-}491}$-FUS^{12E}-fus1$^{792\text{-}1372}$-kanMX-fus1$^{3'UTR}$	Subcloning : pSM2913$^{SalI/NheI}$+pSM2941$^{NotI/XmaI}$	Single integration at endogenous *fus1* locus
pSM3055	pUra4^{PmeI}-p^{nmt1}-fus1-mCherry	Infusion cloning : pSM2602$^{XmaI/SacI}$+pSM684$^{osm8480\text{-}osm8481}$ +pSM2602$^{osm8482\text{-}osm8483}$	Single integration at *ura4*
pSM3056	pUra4^{PmeI}-p^{nmt1}-fus1-mCherry	Infusion cloning : pSM2600$^{XmaI/SacI}$+pSM684$^{osm8484\text{-}osm8481}$ +pSM2602$^{osm8482\text{-}osm8483}$	Single integration at *ura4*
